# Nationwide Screening Unveils Endemic *Ophidiomyces ophidiicola* Presence in Northern Italy, Mainly Affecting Dice Snakes: Evidence from Contemporary and Historical Snake Samples

**DOI:** 10.3390/jof11020118

**Published:** 2025-02-05

**Authors:** Matteo Riccardo Di Nicola, Kevin P. Mulder, Elin Verbrugghe, Federico Storniolo, Naomi Terriere, Luca Colla, Roberto Sacchi, Giacomo Vanzo, Giovanni Zanfei, Daniele Marini, Frank Pasmans, An Martel

**Affiliations:** 1 Wildlife Health Ghent, Faculty of Veterinary Medicine, Ghent University, Salisburylaan 133, 9820 Merelbeke, Belgium; kevin.mulder@ugent.be (K.P.M.); elin.verbrugghe@ugent.be (E.V.); naomi.terriere@ugent.be (N.T.); frank.pasmans@ugent.be (F.P.); an.martel@ugent.be (A.M.); 2 Istituto Zooprofilattico Sperimentale del Piemonte, Liguria e Valle d’Aosta, Via Bologna 148, 10154 Turin, Italy; 3 Asociación Herpetológica Española, Apartado de Correos 191, 28911 Leganés, Spain; 4 Dipartimento di Scienze della Terra e dell’Ambiente, Università degli Studi di Pavia, Via Torquato Taramelli 24, 27100 Pavia, Italy; federico.storniolo01@universitadipavia.it (F.S.); roberto.sacchi@unipv.it (R.S.); giacomo.vanzo01@universitadipavia.it (G.V.); 5 Department of Chemistry, Life Sciences and Environmental Sustainability, University of Parma, Parco Area delle Scienze, 43124 Parma, Italy; luca.colla1@studenti.unipr.it; 6 Department of Life Science, University of Trieste, Via Giorgieri 10, 34127 Trieste, Italy; giovanni.zanfei@phd.units.it; 7 Department of Organismal Biology, Evolutionary Biology Centre, Uppsala University, Norbyvägen 18A, 75236 Uppsala, Sweden; daniele.marini@ebc.uu.se; 8 Department of Veterinary Medicine, University of Perugia, Via San Costanzo 4, 06126 Perugia, Italy

**Keywords:** EIDs, Europe, fungal pathogens, infectious disease, mediterranean, *Natrix*, ophidia, ophidiomycosis, SFD, serpentes

## Abstract

Ophidiomycosis, caused by the keratinophilic fungus *Ophidiomyces ophidiicola* (Oo), is an emerging threat to snake populations, yet its epidemiology in Europe remains underexplored. We investigated the distribution of Oo across free-ranging snake populations in Italy, integrating both recent field samples and historical museum specimens. Our survey involved 423 snakes representing 17 species from 17 regions, with Oo detected in 32 snakes from five different species. Additional molecular detection for *Parananniziopsis* spp. on a subset of 13 Oo-negative samples from snakes that exhibited clinical signs yielded negative results. Acknowledging the non-standardised sampling and the limited sample size, our findings highlight Oo’s persistent and widespread presence across diverse ecological zones, particularly affecting semi-aquatic species like *Natrix tessellata*. While Oo Clade I was primarily found in museum specimens, indicating a historical presence, Clade II prevailed in recent samples. This highlights a complex epidemiological landscape where different clades may influence the current disease dynamics. Our results underscore the importance of continuous surveillance and highlight the need for standardised sampling to better understand snake fungal disease ecology and epidemiology in Italy.

## 1. Introduction

Globally, one-fifth of all reptile species are currently considered endangered [1,2] due to a variety of causes, such as habitat loss and fragmentation, climate change, the pet trade, deliberate persecution, and the introduction of invasive species (e.g., [1,3,4,5,6,7,8,9,10]). The actual threat status of snakes is likely underestimated due to the elusive and evasive nature of many snake species, as well as the general lack of attention directed towards this taxon [3,11,12]. There is evidence of declines in some wild snake populations (e.g., [3,5,9,13,14,15,16]), but data on potential threats in Europe remain scarce, especially with regard to infectious diseases.

Emerging infectious diseases (EIDs), and in particular fungal infections, are a biodiversity concern, since they are capable of rapidly decimating species and population, even leading them to local and global extinction [17,18,19]. For reptiles, most of the fungi associated with disease belong to the Onygenales order, which includes genera such as *Ophidiomyces*, *Emydomyces*, *Nannizziopsis*, and *Paranannizziopsis* [20,21]. Although knowledge on the role of these fungal pathogens as threats for wild snakes in Europe is limited [22], concerns have grown in recent years around ophidiomycosis, also known as snake fungal disease (SFD), caused by the keratinophilic fungus *Ophidiomyces ophidiicola* (Oo; see [23,24,25,26]).

Ophidiomycosis is influenced by various environmental factors such as temperature, moisture, land cover, soil characteristics, and land use, but may vary across different taxa and locations [27,28,29,30,31]. Clinically, ophidiomycosis is characterised by dermatitis, crusts, swelling, and ulceration, leading to increased shedding frequency, dysecdysis, and lethargy [26,32,33]. Shedding can reduce skin lesions, but often does not completely eliminate the fungal presence, resulting in asymptomatic carriers [34,35]. Transmission primarily occurs through direct contact or contaminated substrates, and it is facilitated by interruptions in the epidermis [24,34,36]. However, postnatal transmission from dams to offspring has also been documented [37]. Snakes may change their behaviour following infection, resulting in a fitness reduction [35,38]. For example, some infected snakes increase basking in open areas to elevate body temperature for combating the infection, which in turn increases the risk of predation (e.g., Natricidae; [39]), while other taxa may reduce time spent above ground (e.g., Viperidae; [40]). Some carcasses found outside contaminated hibernacula suggest premature emergence due to infection, leading to death from nighttime frosts [24].

While the full extent of its impact on snake populations remains uncertain [26,39], Oo presence has been documented in snakes across North America, Europe, Asia, and Australia (e.g., [24,41,42,43,44]). Analyses of museum specimens demonstrate that the pathogen has been present in the USA since at least 1945 [45] and in Europe since at least 1959 [46]. Molecular analyses have identified three clades (see [41,42,43,46,47,48,49]): Clade I (“European” clade) was originally isolated from samples in the UK and Czech Republic and is nowadays present only in Europe; Clade II (“North American” clade) has been detected in wild snakes from USA, Europe, and Taiwan and some captive snakes worldwide; Clade III is, so far, only known in a Taiwanese wild snake and in captive snakes. Genomic analyses have revealed that all strains from North American wild snakes belong to Clade II, which likely diverged from Clade I approximately 2000 years ago [47]. Given the relatively high number of positive samples found in captive snakes, the pet trade has been suggested as a potential cause of the spread of different Oo clades across continents [41,44,47].

In Europe, following the first findings of Oo presence reported by Franklinos and colleagues [42], progressively more screenings have been conducted, leading to the current detection of the pathogen in 13 countries and in 11 wild snake species ([12,32,42,46,48,49,50,51,52,53,54,55,56,57,58,59]; see Table S1) out of the 57 present on the continent according to Di Nicola et al. [60]. These findings, however, are still considered preliminary, as there are various European territories where snake populations have not yet been investigated [48]. Italy is one of the most herpetological diverse countries in Europe [61], with 22 species of snakes (Table S2), representing 39% of European snake biodiversity. However, monitoring has, thus far, been limited to a total of 40 wild animals across nine regions, resulting in four positive snakes, all *Natrix tessellata*, from a single location (i.e., Riva del Garda, Lombardy; [12,62]), and therefore, little is known on the full distribution of Oo across Italy.

The primary aim of this study is to increase our knowledge on the distribution and history of Oo and ophidiomycosis in Italy. We collected samples from both wild snakes and museum specimens. For the latter, we limited our screening to species expected to be more susceptible to the pathogen, sampling only specimens that exhibited clinical signs. We conducted both molecular and histological screening and Oo strain identification, linking these findings to the presence of macroscopic skin lesions. These data are essential to direct conservation efforts towards snake species or populations in need and to inform future monitoring efforts through standardised surveys focused on critical areas. It is also an important step for further ecological and epidemiological studies of this emerging infectious disease.

## 2. Materials and Methods

### 2.1. Study Area and Sampling

#### 2.1.1. Wild Snake Sampling

Samples from any free-ranging snakes found in Italy (dead or alive), including the major islands (i.e., Sardinia and Sicily) and the island of Lampedusa, were opportunistically collected during surveys from February 2021 to October 2023. Additionally, previously dated preserved samples, made available by collaborators, were also included. Monitoring areas were focused on sites with known occurrences of threatened taxa or isolated populations (e.g., *Natrix helvetica cetti*, *Vipera ursinii*, *Macroprotodon* cf. *cucullatus*), and based on locations with known positive samples (i.e., large Great Northern Italian Lakes) according to citizen science reports and our previous work [12]. Special occasions were also exploited, such as the “Serpari fests” in Cocullo (see [62,63]) and Pretoro [64], where dozens of “snake hunters” called “serpari” capture wild snakes to display during the festival day. On this occasion, the snakes were kept separately from each other and screened before being shown to the public to prevent cross-contamination.

Following the protocol developed in our previous pilot study [12], each snake was first carefully examined, and potential lesions indicative of fungal infections were photographed. Subsequently, dry swabs were collected from live animals and from some recently road-killed snakes, and skin fragments were collected from the remaining dead snakes and from found skin sheds. Most of the swabs were taken in three replicates, each one consisting of ten repetitions on the dorsal scales, ventral scales, and head region to cover the entire skin surface, followed by ten repetitions on each identified lesion. The swab tips and skin sheds were preserved dry in 1.5 mL tubes and airtight bags, respectively, while tissues from dead specimens were stored frozen dry or placed in 96% ethanol. All samples were stored at −20 °C until processing.

For live snakes, skin fragments for histological investigation were collected only from individuals exhibiting clinical signs with prominent lesions that easily allowed for the removal of small superficial skin fragments while avoiding penetration into the subcutaneous tissue. Scale clipping was performed using disposable sterile surgical scissors (Iris scissors, curved, 11.5 cm; Peha^®^-instrument, Hartmann, Heidenheim an der Brenz, Germany). All tissue samples were then fixed in 10% neutral buffered formalin. Following these procedures, each snake was released at the collection sites.

#### 2.1.2. Museum Specimen Sampling

A retrospective survey was conducted at the Milan Natural History Museum, where all *Natrix helvetica* and *Natrix tessellata* specimens in alcohol collections—stored in separate containers—were visually inspected. These species were selected due to their higher likelihood of yielding positive results for Oo infection (see [12,49]). Only specimens showing gross signs representative of Oo infection were processed. Skin fragments were removed from the areas with lesions using disposable instruments for each specimen, preserved in 96% ethanol, and then frozen.

### 2.2. Molecular Analysis

DNA from swabs was extracted employing the PrepManTM Ultra Sample Preparation Reagent Protocol (Applied Biosystems, Foster City, CA, USA; see [42,65]), including an additional lyticase step (see [66,67]). Specifically, 50 μL of a 6 U/μL lyticase (Cat. No. L4025-25KU; Sigma-Aldrich, St. Louis, MO, USA) solution was added to the swabs and incubated for 1 h at 37 °C in an Eppendorf. After incubation, 50 μL of PrepMan™ Ultra solution was added, and the tube was centrifuged at 8000 rpm for 1 min. The tip with the swab was then removed, and the sample was incubated at 100 °C for 10 min, followed by centrifugation at 13,000 rpm for 3 min. The supernatant, which contained the DNA, was transferred to a new tube for storage and then diluted 10-fold for processing (10 μL DNA with 90 μL HPLC water) to reduce potential inhibition during quantitative PCR.

DNA from sheds and tissues were extracted with DNeasy^®^ Blood and Tissue kit (Qiagen, Inc., Hilden, Germany) with an additional lyticase step. Sheds were first pulverised into a fine powder employing steel balls (20 mm diameter) shaken at 30 Hz for 2 min, and then 50 mg of shed powder was transferred into a 1.5 mL Eppendorf for DNA extraction. Samples were first incubated with lyticase (1 hr at 37 °C with 300 U of lyticase) and proteinase K (overnight at 56 °C), and all subsequent steps were carried out following the manufacturer’s instructions to isolate and purify the DNA.

Molecular detection of Oo was performed by quantitative PCR using primers designed for the intergenic spacer (IGS) region (3′ and 5′ ends) of the ribosomal RNA gene complex (i.e., Oo-rt-IGS-F (forward primer) and Oo-rt-IGS-R (reverse primer)), with a specific probe (Oo-rt-IGS-P; see [68]). This qPCR assay was conducted using the CFX Opus 96 Real-Time PCR System (Bio-Rad, Hercules, CA, USA). Each 25 μL reaction comprised 12.5 μL of 2X IQ supermix (Bio-Rad, USA), 6.25 μL of HPLC water, 0.5 μL of each primer (final concentration of 0.4 μM), 0.25 μL of probe (final concentration at 0.2 μM), and 5 μL of diluted DNA extraction. It entailed an initial denaturation at 95 °C for 3 min, succeeded by 43 cycles of 95 °C for 3 s and 60 °C for 1 min. The qPCR was run in duplicate when at least two swabs were available. For the minority of samples for which only one swab was available (N = 50), we did not run duplicates. Samples were considered positive if at least one replicate gave a positive signal above the qPCR detection threshold of 10 gene copies. Four samples gave a signal below this threshold and gave no signal on the replicate and were, therefore, considered negative in this study.

To identify the clade of *O. ophidiicola* qPCR positive samples, we amplified the same extracted DNA for two short regions (<200 bp) of genes (Actin and ITS) that are known to distinguish between Clade I and Clade II [46]. The PCR conditions for both reactions followed those of Origgi et al. [46], and after visual inspection of the amplification at the expected size on an agarose gel, the successful PCR products were sent for Sanger sequencing in both directions (Eurofins Genomics, Edelsberg, Germany). Resulting sequencing traces were visually inspected and edited in Geneious Prime v2024.0.2 [69], combined with representative data from 15 *O. ophidiicola* strains from GenBank (Table S3), and aligned using MAFFT [70]. The selected regions of both ITS and Actin have two determinative SNPs that correspond with Clade I and Clade II, and these were used to identify the clades of all the sequenced samples. Additionally, to obtain the subclades, the ITS2 sequences were compared with reference *O. ophidiicola* genotypes according to the method used by Blainvillain et al. [48] and Marini et al. [58] (Table S4).

A subset of 13 snakes was also tested for the presence of another pathogenic onygenalean fungus, *Parananniziopsis* sp. This subset primarily consisted of samples that were Oo-negative but exhibited clinical signs (ID: 212, 217, 218, 231, 232, 234, 279, 308, 326, 358, 368, 399, 409).

Detection was carried out following the qPCR protocol described by Lorch et al. [71], using primers and probe targeting the internal transcribed spacer 2 (ITS2). The reaction mixture included 6.5 μL 2X IQ supermix (Bio-Rad, USA), 0.85 μL of HPLC water, 0.26 μL of each primer (final concentration of 0.4 μM), 0.13 μL of probe (final concentration of 0.2 μM), and 5 μL of extracted DNA. The thermocycling program consisted of an initial denaturation at 95 °C for 2 min, followed by 40 cycles of denaturation at 95 °C for 5 s, and annealing/extension at 60 °C for 30 s. A synthetic double-stranded DNA representing the target region was used as a positive control (gBlocksTM Gene Fragments, Integrated DNA Technologies, Coralville, IA, USA).

### 2.3. Histopathology

Histopathological analysis was undertaken to characterise infections and fungal elements. This evaluation was restricted to cases where Oo presence had been confirmed via molecular techniques and when tissue samples were accessible. Tissues were fixed in 10% neutral buffered formalin until they were routinely processed into paraffin blocks and sectioned (5 μm) longitudinally. Slides were subsequently stained with periodic acid–Schiff (PAS) to highlight the fungal components, and then examined under light microscopy.

### 2.4. Statistical Analysis

Prior to statistical analyses, data were filtered to avoid uneven comparisons among taxa. For this purpose, we pooled together sub-specific taxa that belonged to the same species and removed species with insufficient records for robust statistical support. Accordingly, we chose a threshold of 30 records to include species in the models. Additionally, we removed museum records, as they were intentionally selected because clinical signs were detected upon visual inspection and, therefore, could alter the reliability of the models. After this procedure, four snake species were sufficiently represented to be analysed reliably: *H. viridiflavus*, *N. tessellata*, *N. helvetica*, and *V. aspis*. To investigate susceptibility patterns to Oo infection, we performed two Generalised Linear Models (GLMs). In the first GLM, we modelled the probability of a snake to be positive to Oo (thus categorised as a binomial-distributed response variable) according to the presence of visible gross signs and the age of the individual. Furthermore, we also implemented the species in the model to account for different susceptibility patterns. However, due to the opportunistic nature of the sampling, records were not evenly geographically distributed for all species (for example, *N. tessellata* was mainly found in a single population from Garda Lake), making it statistically challenging to separate the effect of the species from that of the locality where it was sampled (i.e., is a species more susceptible to infection or is the pathogen particularly frequent in a specific place?). This means that spatial autocorrelation, where the probability of a snake being positive for Oo depends not only on habitat or life-history traits but also on the occurrence of other positives cases nearby, cannot be excluded. We accounted for this aspect, which mostly affected *N. tessellata*, by performing a second GLM, where, instead, the species, regarded as a proxy of the locality, was implemented as a random intercept (Generalized Linear Mixed effect Model, GLMM). In both models, gross signs and age class were implemented as fixed effects. Analyses were performed in R4.2.2 [72], the GLMM was performed via the “glmer” function of the lme4 R package [73], and visualisation was performed via the visreg R package [74].

## 3. Results

### 3.1. Sampling and Molecular Analysis

Samples were collected from 423 snakes belonging to 17 species (19 taxa, considering the following subspecies of *Natrix helvetica* and *Vipera berus* separately: *N. h. cetti* and *V. b. walser*, due to significant geographical and/or ecological differences), originating from 17 Italian regions. Of these, 304 samples were from free-ranging live snakes, 45 from sheds, 57 from snakes found dead, and 17 from museum specimens (9 *N. helvetica sicula* and 8 *N. tessellata*) preserved at the Milan Natural History Museum with collection dates ranging from 1926 to 1993. Swabs, skin tissue, and sheds were collected for all samples, while additional samples of tissue preserved in formalin for histological evaluation were collected from 28 individuals (see Table S5).

Oo was detected in 32 out of 423 snakes (7.6%) using qPCR (Figure S1). Positive snakes belonged to five different taxa [i.e., *Natrix helvetica sicula* (N = 4), *N. tessellata* (N = 23), *Hierophis viridiflavus* sspp. (N = 3), *Coronella austriaca* (N = 1), and *Vipera aspis francisciredi* (N = 1); Table 1], across 5 of the 17 regions investigated (Table 2). A total of 6 out of the 32 positive samples were museum specimens: four *N. helvetica sicula* (two from Lombardy dated 1964 and 1985, and two from Tuscany both dated 1985) and two *N. tessellata* (from Lombardy dated 1974 and 1985). Excluding the two museum specimens from Tuscany, all remaining positive snake samples originated from regions of Northern Italy (i.e., Piedmont, Lombardy, Veneto, and Trentino-Alto Adige).

One *H. viridiflavus* moult from Sicily with a signal below the detection limit was considered negative. Subsequent amplification and sequencing using the ITS conventional PCR assay resulted in a sequence related to another fungus (unknown sp., with the closest GenBank hit being *Chrysosporium* sp.), which may explain why the qPCR had some low amplification at levels below the detection limit.

**Figure 1:**
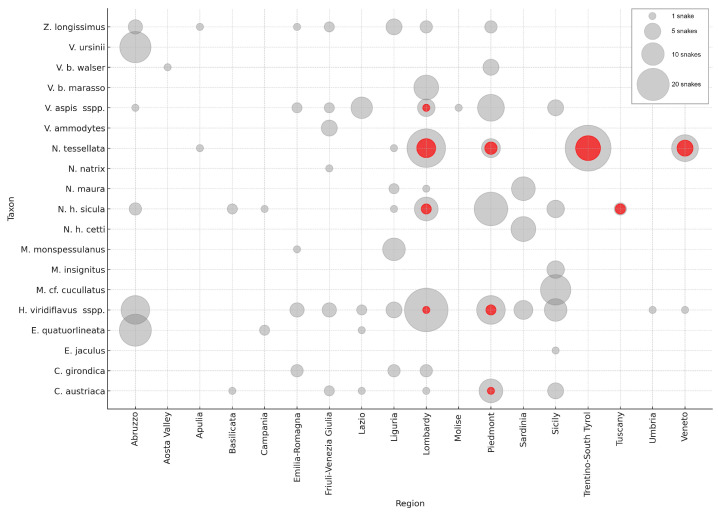
Number of sampled snakes (grey) and Oo-positive snakes (red) by species and region. Data from previous national studies are also included.

Including the records from previous pilot studies conducted in Italy by some of the authors [12,62], the total number of Italian collected samples is currently 463, with 36 of them testing positive for Oo (32 from this study—26 contemporary and 6 museum samples—and 4 additional Oo-positive *N. tessellata* individuals from previous works). The results presented henceforth are based on this combined dataset (Figure 1).

Among the four taxa that included contemporary Oo-positive samples, *N. tessellata* had the highest percent positivity (29.4%, 25 out of 85 samples), followed by *H. viridiflavus* sspp. (2.9%, 3 out of 103 samples), *Coronella austriaca* (4.8%, 1 out of 21 samples), and *V. aspis* (2.5%, 1 out of 40 samples), while all contemporary *Natrix helvetica* spp. samples tested negative (Table 1; Figure S2).

Among the museum specimens, *Natrix tessellata* had a percent positivity of 25% (2 out of 8 samples), and *Natrix helvetica sicula* had a percent positivity of 44.4% (4 out of 9 samples).

The regions with the highest Oo-percent positivity among contemporary snakes were Veneto (35.3%, 5 out of 14 samples), Trentino-Alto Adige (29.3%, 12 out of 41 samples), Lombardy (7.8%, 7 out of 90 samples), and Piedmont (7.7%, 6 out of 78 samples). For the museum samples, the percent positivity values were 66.7% for Tuscany (based on just 2 out of 3 samples) and 30.8% for Lombardy (4 out of 13) (Table 2; Figure S3).

Among the free-ranging snakes, several individuals used in the “serpari fests” of Cocullo and Pretoro, Abruzzo, were also screened (N = 38). Of these, none tested positive for Oo, as found by Marini et al. [62].

**Figure 2:**
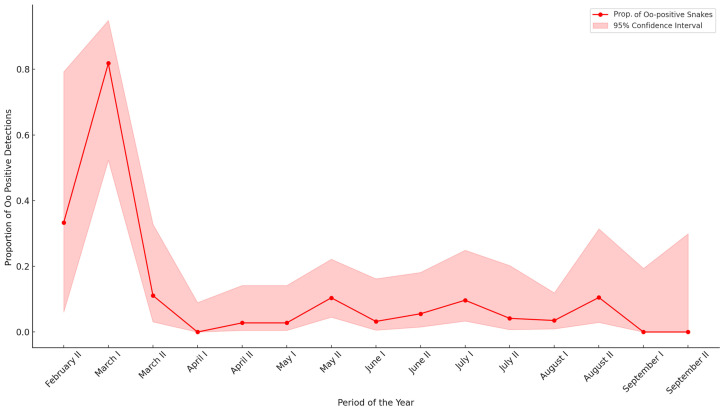
Proportion of Oo-positive snakes among contemporary samples, by period of the year. The x-axis shows the first (I) and second (II) halves of each month starting from February and ending in September. Data from previous national studies are also included.

The time of year with the highest Oo-percent positivity among contemporary samples was the first half of March (81.8%, 9 out of 11 samples). The periods immediately following show notably lower and similar rates, such as the second half of March (11.1%, 2 out of 18 samples), the second half of August (10.5%, 2 out of 19 samples), and the second half of May (10.4%, 5 out of 48 samples) (Figure 2 and Figure S4).

**Figure 3:**
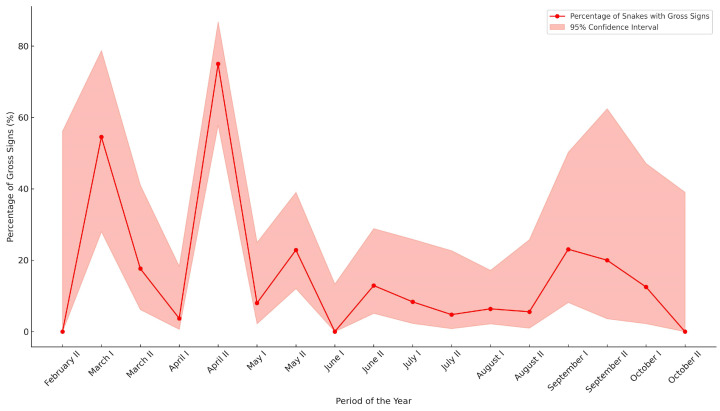
Percentage of snakes with gross signs among contemporary samples, by period of the year. The x-axis shows the first (I) and second (II) halves of each month starting from February and ending in October. Data from previous national studies are also included.

**Figure 4:**
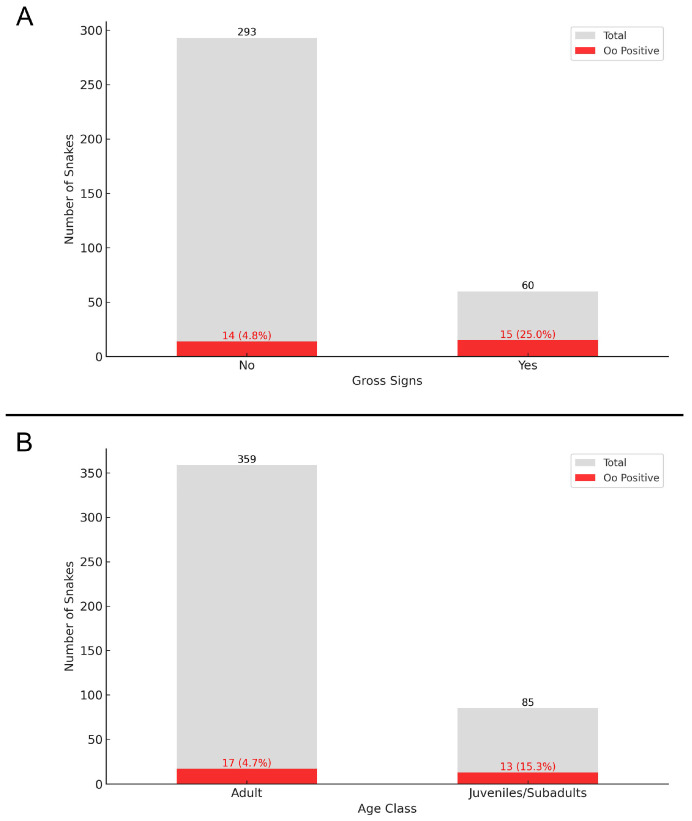
(**A**) Distribution of Oo detection among gross signs presence/absence in contemporary samples (N = 353). (**B**) Distribution of Oo detection among age classes in contemporary samples (N = 444). Red indicates Oo positives, grey Oo negatives. Data from previous national studies are also included.

For 353 contemporary snakes, the presence or absence of gross signs could be recorded. The highest occurrence of gross signs was observed in the second half of April, involving 75% of the snakes (24 out of 32 samples). This was followed by the first half of March, when 54.55% (6 out of 11 samples) exhibited signs. Other periods throughout the year showed significantly lower occurrences of gross signs (Figure 3). Among snakes with gross signs, 25% (15 out of 60 samples) were positive for Oo, of which 14 were *N. tessellata* (93.3%). Conversely, only 4.8% (14 out of 293 samples) of the snakes without gross signs were positive, with 11 being *N. tessellata* (78.6%) (Figure 4A).

The age class was determined for 444 contemporary snakes, including 85 juveniles/subadults, and 359 adults. Juveniles/subadults (15.3%) exhibited a higher percentage of Oo positives compared to adults (4.7%) (Figure 4B).

*Paranannizziopsis* spp. was not detected in all 13 samples in the analysed snake subset.

### 3.2. Clade Determination

Amplification and sequencing were successful for both loci in 13 out of 32 qPCR positive samples, and an additional 9 samples were sequenced for at least one target. Ten qPCR positive samples failed to generate any amplification or clean Sanger sequences. Better amplification success generally correlated with higher gene copy numbers, as found by qPCR (Tables S5 and S6). When both regions were successfully sequenced for the same sample, they always corresponded to the same clade identification. Of the 32 Oo-positive snakes detected in this survey, clade characterisation is, therefore, available for 22.

Clade I was detected in four samples, all from museum collection, specifically two *N. helvetica sicula* (dated 1964 and 1985) and two *N. tessellata* (1974 and 1985), originating from Lombardy. Furthermore, an analysis of the chromatogram for a contemporary *Natrix tessellata* from Veneto revealed the occurrence of a double peak of about equal intensity for two SNPs that distinguish both Clades I and II, indicating a potential co-infection. Notably, Clade II is common in the area but Clade I was also recently found in a *N. tessellata* from the same location in Trentino-Alto Adige [52], indicating that both strains occur in this region.

**Figure 5:**
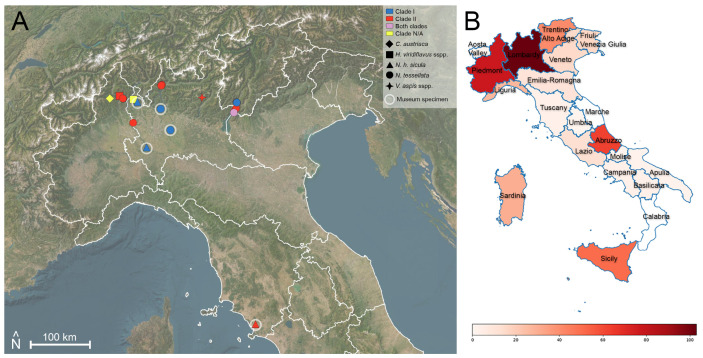
(**A**) Map of central and northern Italy showing the national distribution of Oo-positive samples and Oo clades. Map created using QGIS 3.28 with ESRI Satellite imagery. (**B**) Number of sampled snakes per region in order to visualise sampling effort. Data from our previous national studies are also included.

Clade II was detected in 18 samples from four different taxa (*H. viridiflavus* sspp., *N. h. sicula*, *N. tessellata,* and *V. aspis*), with only one being a museum specimen (*N. h. sicula* from Tuscany, dated 1985). These samples originated from Lombardy, Piedmont, Tuscany, Trentino-Alto Adige, and Veneto (Figure 5). Moreover, the readable ITS2 sequences (N = 14) resulted in three subclades: IA (N = 3), IB (N = 1), and IIDE (N = 10). Details of all processed snake samples, including laboratory analysis results, clades, and subclades can be found in Table S5 and S6. The latter only displays Oo-positive snakes.

### 3.3. Histopathology

**Figure 6:**
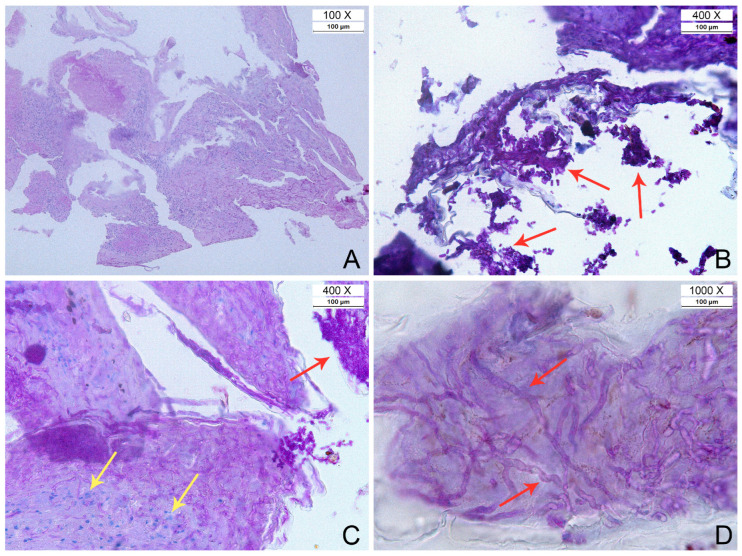
Histological images from selected Oo qPCR positive snakes. (**A**) *Natrix tessellata* (ID 360 in Table S5). Low magnification of necrotic serocellular crust with myriad fungal hyphae within the epidermal necrotic debris and inflammatory elements. (**B**) *Hierophis viridiflavus* (ID 243 in Table S5). Necrotic epidermis showing PAS-positive fungal hyphae and arthroconidial tuft at the air–lesion interface (red arrows). (**C**) *Hierophis viridiflavus* (ID 258 in Table S5). Epidermis with mild spongiosis, transmigrating heterophils (yellow arrows), and multifocal necrosis, with the presence of several hyphae and conidia (red arrow). (**D**) *Hierophis viridiflavus* (ID 258 in Table S5). Fungal hyphae (parallel walls, minimal undulation, occasional transverse septations, non-dichotomous and acute angle branching—highlighted by red arrows) within the epidermal necrotic debris.

Histopathological analysis to characterise infections and fungal elements was performed for nine snakes, all Oo-positive by molecular detection. All the specimens showed signs of inflammation. Fungal elements were detected in only five out of nine samples (three *Natrix tessellata* from Veneto, which showed hyphae; two *Hierophis viridiflavus* sspp. from Piedmont exhibiting hyphae and arthroconidia—see Figure 6). All samples exhibited diverse pathological features and various degrees of necrosis and inflammation, with or without the presence of fungal elements (Figure 6). In some instances, there were small fragments of necrotic epidermis showing full-thickness necrosis with fungal hyphae and arthroconidia consistent with ophidiomycosis. Sometimes, adjacent to these necrotic areas, there were regions of intact epidermis with mild spongiosis and transmigrating heterophils. Additionally, serocellular crusts containing basophilic colonies of bacteria were observed in some samples.

The limited availability of formalin-fixed tissue samples among the Oo-positive cases precluded histological analysis for each, and therefore, a ‘complete’ case classification for all positive snakes was not possible for 23 out of 32 snakes. According to the case classification by Di Nicola et al. [26], we were able to categorise 13 snakes as ’Oo present’, 14 snakes as ’Apparent ophidiomycosis’, 3 snakes as ’Ophidiomycosis’, and 2 snakes as ’Ophidiomycosis and Oo shedder’ (Table 3).

Refer to Tables S5 or S6 for further information regarding individual snakes.

### 3.4. Statistical Analysis

After having thinned the dataset by removing species that were not sufficiently represented and museum specimens, our data consisted of 224 records, including 65 of genus *Hierophis*, 127 of genus *Natrix* (83 of *N. tessellata* and 44 of *N. helvetica*), and 32 of the genus *Vipera* (all of which belonged to *V. aspis*).

The Generalised Linear Model showed a low baseline probability of a snake to be positive for Oo (β = −4.44 ± 0.80, *p* < 0.00001), as well as a positive effect associated with the presence of external gross signs (β = 2.05 ± 0.45, *p* < 0.00001). Additionally, when accounting for species-level effects, we found that *N. tessellata* had a significantly higher probability to be positive compared to other species (β = 2.59 ± 0.78, *p* = 0.0013).

When considering the GLMM, we similarly found a low baseline probability of positives (β = −3.24 ± 0.61, *p* < 0.00001) and a positive effect of the occurrence of gross signs (β = 2.07 ± 0.45, *p* < 0.00001). Concerning the random-effect component, the baseline probabilities differed among species and accounted for 10.2% of the total variance.

**Figure 7:**
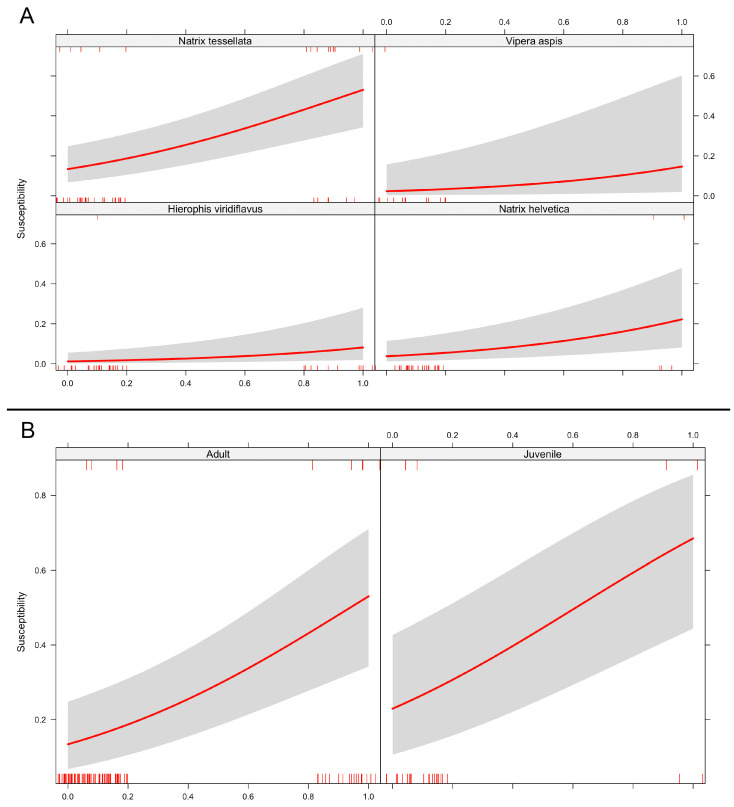
Fixed effects of predictors on the probability of snakes testing positive for Oo infection, implemented in the first GLM. (**A**) The presence of gross signs was significantly correlated with a positive effect on the likelihood of positive detection in all species; the correlation was significantly higher in *N. tessellata* compared to other species. (**B**) No effect was found for the age class.

In both models, no statistically significant effect of age class was found (Figure 7).

## 4. Discussion and Conclusions

Until recently, the presence of *Ophidiomyces ophiidicola* and ophidiomycosis among free-ranging snakes in Italy had been confirmed at only one locality, Northern Lake Garda [12]. The primary aim of this study was, therefore, to gather more information on the extent of Oo presence in Italy. To achieve this, an opportunistic field sampling approach was applied. This strategy, implemented in less than 3 years, allowed for coverage of the majority of Italian regions (Table 2) and involved 423 snakes belonging to most of the Italian species (Table 1). The lack of uniformity in the timing and locations of the field effort limited the creation of a statistically significant overall picture of which regions and species are most affected by Oo and ophidiomycosis. Furthermore, the inconsistency in the sampled/analysed matrices (i.e., not always a swab in triplicate and, in some cases, only tissue samples) may have led to some false negatives. Nonetheless, the survey produced several notable findings that enhance our knowledge about the national situation, enabling more targeted future studies.

We found a species-specific pattern of susceptibility, as *Natrix tessellata* was significantly more frequently positive for Oo compared to other species when considering both statistical models (where, respectively, the taxon was implemented as a fixed or random effect). This is consistent with findings in North American natricids at different latitudes, such as *Nerodia* spp., where ophidiomycosis has been reported with a higher prevalence among certain species [28,75]. Different hypotheses can be formulated to explain this susceptibility. Ecologically, semi-aquatic snakes, like *Natrix* spp., may be more frequently affected by Oo due to the humid environments they inhabit (see [27,49]). Furthermore, natricids are known to engage in mating balls during the reproductive season, which consist of massive aggregation of individuals, mostly males that try to copulate with few females [76,77]. Even though we have limited information and evidence on the horizontal transmission of Oo (see [36]), this reproductive strategy might enhance the probability of contagion. It is likely, as suggested in Northern Pine Snakes (*Pituophis melanoleucus* [78]), that infection in this species primarily occurs through communal den sharing during brumation, leading to contact with contaminated hibernaculum soils [79] or infected individuals. Hence, *N. tessellata* and other natricids might be at higher risk of infection not due to higher physiological susceptibility but rather to their ecological features, which render natricids an interesting case study to further investigate ecological determinants of infection patterns.

Seasonal variations in Oo presence are influenced by host ecology and environmental conditions, affecting pathogen detection rates. The disease predominantly occurs during the spring months when snakes emerge from hibernation and are more susceptible due to the reactivation of their metabolic functions [26,49]. However, Joudrier et al.’s analysis for Europe did not find a significant seasonal association with disease incidence, suggesting that other ecological factors may play a more pivotal role in influencing infection rates [49]. Our findings suggest an increased prevalence of the pathogen in early March. However, the opportunistic nature of the sampling limits the ability to conduct a consistent analysis throughout the active months of snakes. Acknowledging this bias, we identified significant associations between Oo positivity and the presence of cutaneous gross signs. As shown in Figure 3, not only the second half of March but also, more significantly, the second half of April is the period with the highest percentage of snakes exhibiting gross signs. This seasonal discrepancy in Oo positives and gross signs was influenced by the predominance of samples in the second half of April from the study by Marini and colleagues [62]. These samples, collected during Cocullo’s ’serpari’ festival, represent a subset selected exclusively for their gross signs, but all were found to be Oo-negative.

Geographically, all positives from contemporary samples were recorded in northern regions (i.e., Piedmont, Lombardy, Veneto, and Trentino-Alto Adige), with 24 out of the 30 overall contemporary positives being *N. tessellata* from the shores of the major North Italian lakes, such as Lake Garda (N = 17), Lake Como (N = 4), and Lake Maggiore (N = 3). Additionally, two museum samples from Tuscany tested positive, suggesting the potential presence of Oo at lower latitudes across the national territory. This indicates the need for further field efforts in central and southern Italy, focusing on areas with significant populations of *Natrix* spp. in lacustrine zones, such as the three major lakes of central Italy—Lake Bolsena, Lake Trasimeno, and Lake Bracciano—where the presence of *N. tessellata* is well-documented but from which no samples have yet been collected and no reports of snakes with clinical signs have been received, even from citizen science.

Following molecular clade determination, we found that both Clade I and Clade II occur in Italy. Clade I was detected mainly in museum collection specimens (4 out of 5 samples), whereas Clade II occurred most frequently in recent samples (16 out of 17 samples). Additionally, we found an interesting case from Garda Lake that is possibly positive for both clades, suggesting the potential for co-infection, as observed in other areas by Blanvillain et al. [48]. Although the limited sampling does not allow to conclusively infer the historical dynamics of Oo in Northern Italy, it appears that Clade I has been in Northern Italy for at least sixty years and was likely the main clade present historically. The high rate of Clade II in modern samples (94%) suggests that this is now the dominant clade and that it might have outcompeted Clade I. This could be related to the faster growth rate of this clade, as found by Franklinos et al. [42]. However, the timing of co-occurrence of both clades in wild populations from Italy cannot be accurately reconstructed without more data and requires more extensive investigations at larger spatial and temporal scales.

In conclusion, we report a pattern of infection spread across Northern Italy worthy of attention. Several species were found to be Oo-positive, and its presence is not confined to single localities, which suggests that ophidiomycosis might occur at a much wider range than was previously known. Ongoing monitoring, through the implementation of standardised surveys, will be crucial to enhancing our understanding of the ecology of *O. ophidiicola* and the disease across Italy.

## Figures and Tables

**Table 1:** Number of snakes sampled and percentage of Oo positives per snake taxon. Overall results include previous national studies [12,62]. For *Natrix helvetica sicula*, *N. tessellata*, and the total, the data are divided into contemporary (left) and museum (right) samples.

	**Present Study**		**Overall Results**	
**Snake Taxon**	**N° of Snakes**	**% Oo Positives**	**N° of Snakes**	**% Oo Positives**
*Coronella austriaca*	21	4.8%	21	4.8%
*Coronella girondica*	6	0%	9	0%
*Elaphe quatuorlineata*	10	0%	23	0%
*Eryx jaculus*	1	0%	1	0%
*Hierophis viridiflavus* sspp.	90	3.3%	103	2.9%
*Macroprotodon* cf. *cucullatus*	18	0%	18	0%
*Malpolon insignitus*	6	0%	6	0%
*Malpolon monspessulanus*	11	0%	11	0%
*Natrix helvetica cetti*	11	0%	12	0%
*Natrix helvetica sicula*	37|9	0%|44.4%	40|9	0%|44.4%
*Natrix maura*	14	0%	14	0%
*Natrix natrix vulgaris*	1	0%	1	0%
*Natrix tessellata*	81|8	25.9%|25%	85|8	29.4%|25%
*Vipera ammodytes*	5	0%	5	0%
*Vipera aspis* sspp.	40	2.5%	40	2.5%
*Vipera berus marasso*	12	0%	12	0%
*Vipera berus walser*	6	0%	6	0%
*Vipera ursinii*	19	0%	19	0%
*Zamenis longissimus*	16	0%	19	0%
Unlabelled sample	1	0%	1	0%
**Total**	406|17	6.4%|35.3%	446|17	6.7%|35.3%

**Table 2:** Number of snakes sampled and percentage of Oo positives per region. Overall results include previous national studies [12,62]. For Lombardy, Tuscany, and Veneto, the data are divided into contemporary (left) and museum (right) samples.

	**Present Study**		**Overall Results**	
**Region**	**N° of Snakes**	**% Oo Positives**	**N° of Snakes**	**% Oo Positives**
Abruzzo	40	0%	63	0%
Aosta Valley	1	0%	1	0%
Apulia	2	0%	2	0%
Basilicata	3	0%	3	0%
Campania	3	0%	3	0%
Emilia-Romagna	9	0%	11	0%
Friuli-Venezia Giulia	16	0%	16	0%
Lazio	12	0%	13	0%
Liguria	26	0%	27	0%
Lombardy	84|13	8.3%|30.8%	90|13	7.8%|30.8%
Molise	1	0%	1	0%
Piedmont	77	7.8%	78	7.7%
Sardinia	29	0%	30	0%
Sicily	51	0%	51	0%
Trentino-Alto Adige/Südtirol	37	21.6%	41	29.3%
Tuscany	0|3	0|66.7%	0|3	0|66.7%
Umbria	0	0%	1	0%
Veneto	14|1	35.7%|0%	14|1	35.7%|0%
Unlabelled sample	1	0%	1	0%
**Total**	406|17	8.3%|30.8%	446|17	6.7%|35.3%

**Table 3:** Case classification of the sampled snakes. The overall results include previous national studies [12,62].

**Case Classification**	**Gross Signs**	**Molecular** **Detection**	**Hyphae** **(Histology)**	**Arthroconidia (Histology)**	**Present Study Results**	**Overall Results**
Oo present	N or N/A	Y	N or N/A	N or N/A	13	14
Apparent ophidiomycosis	Y	Y	N or N/A	N or N/A	14	14
Ophidiomycosis	Y or N/A	Y	Y	N	3	4
Ophidiomycosis and Oo shedder	Y or N/A	Y	Y	Y	2	4

## Data Availability

All data generated or analysed during this study are included in this published article and its Supplementary Information Files.

## Supplementary Materials

The following supporting information can be downloaded at https://www.mdpi.com/article/10.3390/jof11020118/s1, Table S1: Positive detections of Ophidiomyces ophidiicola in Europe; Table S2: List of species and subspecies of Italian snakes. From Di Nicola et al. [80]; Table S3: Clade determination; Table S4: Subclade determination; Table S5: List of snakes considered in the study, including 423 animals from the current survey and 40 from our previous studies. Coordinates are approximate for conservation purposes. Oo-positive snakes are highlighted in yellow. Oo case classification is based on Di Nicola et al. [26]; Table S6: List of Oo-positive snakes only, including 32 animals from the current survey and 4 from our previous studies; Figure S1: Map of Italy with the sampling points of the 423 snakes included in the present survey. Locations with snakes negative for Oo molecular detection are marked in grey, while locations with positive snakes are marked in red. Map created using QGIS 3.28 with ESRI Satellite imagery; Figure S2: Number of sampled snakes (gray) and Oo-positive cases (red) by snake taxon. Data from our previous national studies are also included; Figure S3: Number of sampled snakes (gray) and Oo-positive cases (red) by region. Data from our previous national studies are also included; Figure S4: Number of sampled snakes (gray) and Oo-positive cases (red) by time of year. The x-axis shows the first (I) and second (II) halves of each month. Data from our previous national studies are also included.
