# Supplementary material for: Nationwide Screening Unveils Endemic Ophidiomyces ophidiicola Presence in Northern Italy, Mainly Affecting Dice Snakes: Evidence from Contemporary and Historical Snake Samples
Source: J Fungi (Basel). 2025 Feb 5;11(2):118. doi: 10.3390/jof11020118 (PMC11856666; doi:10.3390/jof11020118)
Supplement: Supplementary file 1 [file jof-11-00118-s001.zip › jof-3443492-supplementary.pdf]

Nationwide Screening Unveils Endemic *Ophidiomyces ophidiicola* Presence in Northern Italy, Mainly Affecting Dice Snakes: Evidence from Contemporary and Historical Snake Samples

Matteo Riccardo Di Nicola, Kevin P. Mulder, Elin Verbrugghe, Federico Storniolo, Naomi Terriere, Luca Colla, Roberto Sacchi, Giacomo Vanzo, Giovanni Zanfei, Daniele Marini, Frank Pasmans, An Martel

SUPPLEMENTARY MATERIAL

Table S1. Positive detections of *Ophidiomyces ophidiicola* in Europe.

**Reference**	**Oo positive species**	**EU Countries with Oo positives**	**Oo clades identified**
Franklinos et al., 2017 [42]	*Natrix helvetica*, *Natrix tessellata*, *Vipera berus*	United Kingdom; Czechia	I: Czechia, United Kingdom
Meier et al., 2018 [32]	*Natrix helvetica*	Switzerland	N/A
Schüler et al., 2022 [50]	*Natrix tessellata*	Germany	N/A
Origgi et al., 2022 [46]	*Natrix helvetica*, *Natrix natrix*, *Natrix tessellata*	Italy; Switzerland	I: Italy, Switzerland<br>II: Switzerland
Marini et al., 2023° [12]<br>+<br>Marini et al., 2023b [52]	*Natrix tessellata*	Italy	I
Přibyl et al., 2023 [53]	*Coronella austriaca*, *Natrix natrix*, *Natrix tessellata*, *Zamenis longissimus*	Czechia; Slovakia	N/A
Schüler et al., 2024 [51]	*Natrix tessellata*	Germany	N/A
Joudrier et al., 2024 [49]	*Natrix tessellata*, *Natrix helvetica*, *Natrix maura*, *Hierophis viridiflavus*	Switzerland	I; II
Allain et al., 2024 [54]	*Natrix helvetica*	United Kingdom	N/A
Blanvillain et al., 2024 [48]	*Coronella austriaca*, *Hierophis viridiflavus*, *Natrix helvetica*, *Natrix maura*, *Natrix natrix*, *Natrix tessellata*, *Vipera berus*, *Vipera nikolski*, *Zamenis longissimus*	Austria, Czechia, France, Germany, Hungary, Poland, Switzerland, Ukraine	I: Austria, Czechia, Germany, Hungary, Poland, Switzerland, Ukraine,<br>II: Czechia, France, Switzerland, Ukraine
Stark et al., 2024 [55]	*Natrix helvetica*	The Netherlands	N/A
Joudrier et al., 2024 [56]	*Vipera aspis*	Switzerland	N/A
Martinez-Silvestre et al. 2024a [57]	*Zamenis longissimus*	Spain	II
Marini et al. 2024 [58]	*Zamenis longissimus*	Poland	I

Martinez-Silvestre et al. 2024b [59]	*Coronella girondica, Zamenis longissimus*	Spain	N/A
This study	*Coronella austriaca, Hierophis viridiflavus, Natrix helvetica, Natrix tessellata, Vipera aspis*	Italy	I; II

Table S2. List of species and subspecies of Italian snakes. From Di Nicola et al. [80].

**Family**	**Species**	**Subspecies**	**Figure**
Typhlopidae Merrem 1820	*Indotyphlops braminus* (Daudin 1803)	*Indotyphlops braminus* (Daudin 1803)	S1 A
Erycidae Bonaparte 1840	*Eryx jaculus* (Linnaeus 1758)	*Eryx jaculus* (Linnaeus 1758) subspecific framework to be defined	S1 B
Psammophiidae Boie 1827	*Malpolon insignitus* (Geoffroy Saint-Hilaire 1827)	*Malpolon insignitus insignitus* (Geoffroy Saint-Hilaire 1827)	S2 A
	Malpolon monspessulanus (Hermann 1804)	*Malpolon monspessulanus monspessulanus* (Hermann 1804)	S2 B
Natricidae Bonaparte 1840	*Natrix helvetica* (Lacépède 1789)	*Natrix helvetica cetti* Gené 1839	S2 C
		Natrix helvetica sicula (Cuvier 1829)	S2 D
	Natrix maura (Linnaeus 1758)	*Natrix maura* (Linnaeus 1758) monotypic	S2 E
	Natrix natrix (Linnaeus 1758)	*Natrix natrix vulgaris* Laurenti 1768	S2 F
	Natrix tessellata (Laurenti 1768)	*Natrix tessellata* (Laurenti 1768) monotypic	S2 G
Colubridae Oppel 1811	*Coronella austriaca* Laurenti 1768	*Coronella austriaca austriaca* Laurenti 1768	S3 A
	Coronella girondica (Daudin 1803)	*Coronella girondica* (Daudin 1803) monotypic	S3 B
	Elaphe quatuorlineata (Bonnaterre 1790)	*Elaphe quatuorlineata quatuorlineata* (Bonnaterre 1790)	S3 C
	Hemorrhois hippocrepis (Linnaeus 1758)	*Hemorrhois hippocrepis* (Linnaeus 1758) monotypic	S3 D
	Hierophis viridiflavus (Lacépède 1789)	*Hierophis viridiflavus viridiflavus* (Lacépède 1789)	S3 E
		Hierophis viridiflavus carbonarius (Bonaparte 1833)	S3 F
	Macroprotodon cf. *cucullatus* (Geoffroy Saint-Hilaire 1827)	*Macroprotodon* cf. *cucullatus* (Geoffroy Saint-Hilaire 1827)	S3 G
	Telescopus fallax (Fleischmann 1831)	*Telescopus fallax fallax* (Fleischmann 1831)	S4 A
	Zamenis lineatus (Camerano 1891)	*Zamenis lineatus* (Camerano 1891) monotypic	S4 B
	Zamenis longissimus (Laurenti 1768)	*Zamenis longissimus* (Laurenti 1768) monotypic	S4 C
	Zamenis situla (Linnaeus 1758)	*Zamenis situla* (Linnaeus 1758) monotypic	S4 D
Viperidae Oppel 1811	*Vipera ammodytes* (Linnaeus 1758)	*Vipera ammodytes ammodytes* (Linnaeus 1758)	2 A
	Vipera aspis (Linnaeus 1758)	*Vipera aspis aspis* (Linnaeus 1758)	2 B

		Vipera aspis francisciredi (Laurenti 1768)	2 C
		Vipera aspis hugyi (Schinz 1834)	2 D
	Vipera berus (Linnaeus 1758)	*Vipera berus marasso* (Pollini 1818)	2 E
		Vipera berus walser Ghielmi, Menegon, Marsden, Laddaga & Ursenbacher 2016	2 F
	Vipera ursinii (Bonaparte 1835)	*Vipera ursinii ursinii* (Bonaparte 1835)	2 G

Table S3. Clade determination.

Clade	Strain	Source	GenBank ITS	GenBank Actin
Clade I	NWHC 45692-02	[42]	KY474059	KY474070
Clade I	NWHC 45692-12	[42]	KY474060	KY474071
Clade I	NWHC 45707-81	[42]	KY474061	KY474072
Clade I	NWHC 45707-82	[42]	KY474062	KY474073
Clade I	NWHC 45707-83	[42]	KY474063	KY474074
Clade I	NWHC 45707-84	[42]	KY474064	KY474075
Clade II	CGMHD 2664-1	[43]	MT906449	MT912509
Clade II	CGMHD 2664-2	[43]	MT906445	MT912510
Clade II	NWHC 23942-01	[42]	KY474065	KY474076
Clade II	UAMH 10769	[42]	KF477235	KY474069
Clade II	UAMH 6218	[42]	KF477227	KY474066
Clade II	UAMH 6642	[42]	KC884267	KY474067
Clade III	CGMHD 2605-2	[43]	MT905070	MT912507
Clade III	CGMHD 2605-3	[43]	MT906448	MT912508
Clade III	UAMH 6688	[42]	KF477228	KY474078

Table S4. Subclade determination.

Clade	Strain	Source	GenBank ITS
Clade II-DE	NWHC 27242-2	[48]	OL457490
Clade II-F	NWHC 24281-01	[48]	KX148658
Clade I-A	NWHC 45692-02	[48]	KY474059
Clade I-B	NWHC 45707-81	[48]	KY474061

Table S5. List of snakes considered in the study, including 423 animals from the current survey and 40 from our previous studies. Coordinates are approximate for conservation purposes. Oo positive snakes are highlighted in yellow. Oo case classification is based on Di Nicola et al. [26].

ID	Taxon	Date	Lat.	Long.	Region	Age class	Gross signs	Sample type	Oo mol. detection	Copies Average of above 10	Oo Clade (subclade)	Histology	Histology report	Case classification	Notes	Refs.
1	*Coronella girondica*	2021-10-05	44.4	11.3	Emilia-Romagna	Juvenile	Yes	Tissue (in alcohol)	Negative	N/A	N/A	No	N/A	N/A	Found dead	[12]
2	*Coronella girondica*	2021-11-11	44.4	9.0	Liguria	Subadult	No	Tissue (frozen)	Negative	N/A	N/A	No	N/A	N/A	Found dead	[12]
3	*Coronella girondica*	2021-02-02	43.9	11.9	Emilia-Romagna	Adult	No	Dry swab (3)	Negative	N/A	N/A	No	N/A	N/A		[12]
4	*Hierophis viridiflavus* sspp.	2021-05-10	45.3	9.2	Lombardy	Subadult	No	Tissue (frozen)	Negative	N/A	N/A	No	N/A	N/A	Found dead	[12]
5	*Hierophis viridiflavus* sspp.	2021-09-29	42.0	12.7	Lazio	Adult	Yes	Tissue (in alcohol)	Negative	N/A	N/A	No	N/A	N/A	Found dead	[12]
6	*Hierophis viridiflavus* sspp.	2021-05-13	43.0	12.4	Umbria	Juvenile	Yes	Dry swab (3)	Negative	N/A	N/A	No	N/A	N/A		[12]
7	*Hierophis viridiflavus* sspp.	2021-05-19	46.2	9.4	Lombardy	Adult	Yes	Dry swab (3)	Negative	N/A	N/A	No	N/A	N/A		[12]
8	*Hierophis viridiflavus* sspp.	2021-02-09	44.9	10.9	Lombardy	Adult	No	Tissue (frozen)	Negative	N/A	N/A	No	N/A	N/A	Found dead	[12]
9	*Hierophis viridiflavus* sspp.	2021-03-10	45.2	9.4	Lombardy	Adult	No	Tissue (in alcohol)	Negative	N/A	N/A	No	N/A	N/A	Found dead	[12]
10	*Natrix helvetica cetti*	2021-04-03	39.3	9.4	Sardinia	Adult	No	Dry swab (3)	Negative	N/A	N/A	No	N/A	N/A		[12]
11	*Natrix helvetica sicula*	2021-09-01	45.7	9.4	Lombardy	Subadult	No	Tissue (in alcohol)	Negative	N/A	N/A	No	N/A	N/A	Found dead	[12]
12	*Natrix helvetica sicula*	2021-04-25	45.1	7.5	Piedmont	Subadult	No	Dry swab (3)	Negative	N/A	N/A	No	N/A	N/A		[12]
13	*Natrix helvetica sicula*	2021-07-01	45.3	9.2	Lombardy	Adult	No	Tissue (frozen)	Negative	N/A	N/A	No	N/A	N/A	Found dead	[12]
14	*Natrix tessellata*	2021-03-02	45.9	10.9	Trentino-South Tyrol	Juvenile	Yes	Dry swab (3) + Tissue (in alcohol)	Positive	N/A	I	Yes	Hyphae and arthroconidia	Ophidiomycosis and Oo shedder		[12,52]
15	*Natrix tessellata*	2021-03-02	45.9	10.9	Trentino-South Tyrol	Juvenile	Yes	Dry swab (3) + Tissue (in alcohol)	Positive	N/A	N/A	Yes	Hyphae	Ophidiomycosis		[12]
16	*Natrix tessellata*	2021-03-02	45.9	10.9	Trentino-South Tyrol	Juvenile	Yes	Dry swab (3) + Tissue (in alcohol)	Positive	N/A	N/A	Yes	Hyphae and arthroconidia	Ophidiomycosis and Oo shedder		[12]
17	*Natrix tessellata*	2021-03-02	45.9	10.9	Trentino-South Tyrol	Adult	No	Dry swab (3) + Tissue (in alcohol)	Positive	N/A	N/A	No	N/A	Oo present		[12]
18	*Elaphe quatuorlineata*	2019-04-29	42.0	13.8	Abruzzo	Subadult	Yes	Dry swab (1)	Negative	N/A	N/A	No	N/A	N/A	Cocullo Serpari fest	[62]

19	*Hierophis viridiflavus* sspp.	2019-04-29	42.0	13.8	Abruzzo	Adult	Yes	Dry swab (1)	Negative	N/A	N/A	No	N/A	N/A	Cocullo Serpari fest	[62]
20	*Elaphe quatuorlineata*	2019-04-29	42.0	13.8	Abruzzo	Adult	Yes	Dry swab (1)	Negative	N/A	N/A	No	N/A	N/A	Cocullo Serpari fest	[62]
21	*Hierophis viridiflavus* sspp.	2019-04-29	42.0	13.8	Abruzzo	Adult	Yes	Dry swab (1)	Negative	N/A	N/A	No	N/A	N/A	Cocullo Serpari fest	[62]
22	*Hierophis viridiflavus* sspp.	2019-04-29	42.0	13.8	Abruzzo	Adult	Yes	Dry swab (1)	Negative	N/A	N/A	No	N/A	N/A	Cocullo Serpari fest	[62]
23	*Elaphe quatuorlineata*	2019-04-29	42.0	13.8	Abruzzo	Adult	Yes	Dry swab (1)	Negative	N/A	N/A	No	N/A	N/A	Cocullo Serpari fest	[62]
24	*Zamenis longissimus*	2019-04-29	42.0	13.8	Abruzzo	Adult	Yes	Dry swab (1)	Negative	N/A	N/A	No	N/A	N/A	Cocullo Serpari fest	[62]
25	*Zamenis longissimus*	2019-04-29	42.0	13.8	Abruzzo	Adult	Yes	Dry swab (1)	Negative	N/A	N/A	No	N/A	N/A	Cocullo Serpari fest	[62]
26	*Hierophis viridiflavus* sspp.	2019-04-29	42.0	13.8	Abruzzo	Adult	Yes	Dry swab (1)	Negative	N/A	N/A	No	N/A	N/A	Cocullo Serpari fest	[62]
27	*Elaphe quatuorlineata*	2019-04-29	42.0	13.8	Abruzzo	Adult	Yes	Dry swab (1)	Negative	N/A	N/A	No	N/A	N/A	Cocullo Serpari fest	[62]
28	*Elaphe quatuorlineata*	2019-04-29	42.0	13.8	Abruzzo	Adult	Yes	Dry swab (1)	Negative	N/A	N/A	No	N/A	N/A	Cocullo Serpari fest	[62]
29	*Elaphe quatuorlineata*	2019-04-29	42.0	13.8	Abruzzo	Adult	Yes	Dry swab (1) + Tissue (frozen)	Negative	N/A	N/A	Yes	Negative	N/A	Cocullo Serpari fest	[62]
30	*Elaphe quatuorlineata*	2019-04-29	42.0	13.8	Abruzzo	Adult	Yes	Dry swab (1)	Negative	N/A	N/A	No	N/A	N/A	Cocullo Serpari fest	[62]
31	*Elaphe quatuorlineata*	2019-04-29	42.0	13.8	Abruzzo	Adult	Yes	Dry swab (1)	Negative	N/A	N/A	No	N/A	N/A	Cocullo Serpari fest	[62]
32	*Elaphe quatuorlineata*	2019-04-29	42.0	13.8	Abruzzo	Adult	Yes	Dry swab (1)	Negative	N/A	N/A	No	N/A	N/A	Cocullo Serpari fest	[62]
33	*Hierophis viridiflavus* sspp.	2019-04-29	42.0	13.8	Abruzzo	Adult	Yes	Dry swab (1)	Negative	N/A	N/A	No	N/A	N/A	Cocullo Serpari fest	[62]
34	*Hierophis viridiflavus* sspp.	2019-04-29	42.0	13.8	Abruzzo	Adult	Yes	Dry swab (1)	Negative	N/A	N/A	No	N/A	N/A	Cocullo Serpari fest	[62]
35	*Hierophis viridiflavus* sspp.	2019-04-29	42.0	13.8	Abruzzo	Adult	Yes	Dry swab (1) + Tissue (frozen)	Negative	N/A	N/A	No	N/A	N/A	Cocullo Serpari fest	[62]
36	*Zamenis longissimus*	2019-04-29	42.0	13.8	Abruzzo	Adult	Yes	Dry swab (1) + Tissue (frozen)	Negative	N/A	N/A	No	N/A	N/A	Cocullo Serpari fest	[62]
37	*Elaphe quatuorlineata*	2019-04-30	42.0	13.8	Abruzzo	Adult	Yes	Dry swab (1)	Negative	N/A	N/A	No	N/A	N/A	Cocullo Serpari fest	[62]
38	*Elaphe quatuorlineata*	2019-04-30	42.0	13.8	Abruzzo	Adult	Yes	Dry swab (1)	Negative	N/A	N/A	No	N/A	N/A	Cocullo Serpari fest	[62]
39	*Elaphe quatuorlineata*	2019-04-30	42.0	13.8	Abruzzo	Adult	Yes	Dry swab (1)	Negative	N/A	N/A	No	N/A	N/A	Cocullo Serpari fest	[62]

40	*Elaphe quatuorlineata*	2019-04-30	42.0	13.8	Abruzzo	Adult	Yes	Dry swab (1)	Negative	N/A	N/A	No	N/A	N/A	Cocullo Serpari fest	[62]
41	*Hierophis viridiflavus* sspp.	2022-06-22	46.2	13.0	Friuli-Venezia Giulia	Adult	N/A	Molt (frozen)	Negative	N/A	N/A	No	N/A	N/A	Shed	This study
42	*Vipera ammodytes*	2022-06-23	45.8	13.5	Friuli-Venezia Giulia	Adult	N/A	Molt (frozen)	Negative	N/A	N/A	No	N/A	N/A	Shed	This study
43	*Vipera ammodytes*	2022-06-20	45.8	13.5	Friuli-Venezia Giulia	Adult	No	Dry Swab (3)	Negative	N/A	N/A	No	N/A	N/A		This study
44	*Zamenis longissimus*	2022-06-20	45.8	13.6	Friuli-Venezia Giulia	Adult	No	Dry Swab (3)	Negative	N/A	N/A	No	N/A	N/A	Found dead	This study
45	*Natrix natrix*	2022-06-21	46.2	12.5	Friuli-Venezia Giulia	Adult	No	Dry Swab (3)	Negative	N/A	N/A	No	N/A	N/A		This study
46	*Coronella austriaca*	2022-06-21	46.2	12.5	Friuli-Venezia Giulia	Adult	No	Dry Swab (3)	Negative	N/A	N/A	No	N/A	N/A		This study
47	*Coronella austriaca*	2022-06-21	46.2	12.5	Friuli-Venezia Giulia	Adult	No	Dry Swab (3)	Negative	N/A	N/A	No	N/A	N/A		This study
48	*Hierophis viridiflavus* sspp.	2021-08-17	44.7	9.4	Emilia-Romagna	Adult	No	Dry Swab (3)	Negative	N/A	N/A	No	N/A	N/A		This study
49	*Vipera ursinii*	2021-08-06	42.4	13.6	Abruzzo	Adult	No	Dry Swab (3)	Negative	N/A	N/A	No	N/A	N/A		This study
50	*Vipera ursinii*	2021-08-06	42.4	13.6	Abruzzo	Adult	No	Dry Swab (3)	Negative	N/A	N/A	No	N/A	N/A		This study
51	*Vipera ursinii*	2021-08-06	42.4	13.6	Abruzzo	Adult	No	Dry Swab (3)	Negative	N/A	N/A	No	N/A	N/A		This study
52	*Vipera aspis* sspp.	2022-08-07	41.7	14.0	Molise	Juvenile	Yes	Dry Swab (3)	Negative	N/A	N/A	No	N/A	N/A		This study
53	*Natrix helvetica sicula*	2021-07-26	45.1	7.5	Piedmont	Subadult	No	Dry Swab (3)	Negative	N/A	N/A	No	N/A	N/A		This study
54	*Vipera aspis* sspp.	2021-08-08	41.7	13.9	Lazio	Adult	No	Dry Swab (3)	Negative	N/A	N/A	No	N/A	N/A		This study
55	*Vipera aspis* sspp.	2021-08-08	41.7	13.9	Lazio	Adult	No	Dry Swab (3)	Negative	N/A	N/A	No	N/A	N/A		This study
56	*Vipera aspis* sspp.	2021-09-05	45.7	7.9	Piedmont	Adult	No	Dry Swab (3)	Negative	N/A	N/A	No	N/A	N/A		This study
57	*Hierophis viridiflavus* sspp.	2022-07-30	43.9	11.8	Emilia-Romagna	Adult	No	Dry Swab (3)	Negative	N/A	N/A	No	N/A	N/A		This study
58	*Vipera ursinii*	2022-08-02	42.4	13.6	Abruzzo	Adult	No	Dry Swab (3)	Negative	N/A	N/A	No	N/A	N/A		This study
59	*Vipera ursinii*	2022-08-02	42.4	13.6	Abruzzo	Adult	No	Dry Swab (3)	Negative	N/A	N/A	No	N/A	N/A		This study
60	*Vipera ursinii*	2022-08-02	42.4	13.6	Abruzzo	Adult	No	Dry Swab (3)	Negative	N/A	N/A	No	N/A	N/A		This study

61	*Vipera aspis* sspp.	2022-08-08	41.7	13.9	Lazio	Adult	No	Dry Swab (3)	Negative	N/A	N/A	No	N/A	N/A		This study
62	*Vipera aspis* sspp.	2022-08-08	41.7	13.9	Lazio	Adult	No	Dry Swab (3)	Negative	N/A	N/A	No	N/A	N/A		This study
63	*Vipera aspis* sspp.	2022-08-08	41.7	13.9	Lazio	Adult	No	Dry Swab (3)	Negative	N/A	N/A	No	N/A	N/A		This study
64	*Vipera aspis* sspp.	2022-08-12	41.7	13.9	Lazio	Adult	No	Dry Swab (3)	Negative	N/A	N/A	No	N/A	N/A		This study
65	*Vipera aspis* sspp.	2022-07-31	43.9	11.8	Emilia-Romagna	Juvenile	No	Dry Swab (3)	Negative	N/A	N/A	No	N/A	N/A		This study
66	*Vipera ursinii*	2022-08-01	42.4	13.7	Abruzzo	Adult	No	Dry Swab (3)	Negative	N/A	N/A	No	N/A	N/A		This study
67	*Vipera aspis* sspp.	2022-08-01	42.4	13.7	Abruzzo	Adult	No	Dry Swab (3)	Negative	N/A	N/A	No	N/A	N/A		This study
68	*Hierophis viridiflavus* sspp.	2022-08-08	41.7	13.9	Lazio	Adult	No	Dry Swab (3)	Negative	N/A	N/A	No	N/A	N/A	Found dead	This study
69	*Vipera ursinii*	2022-08-05	42.4	13.6	Abruzzo	Adult	No	Dry Swab (3)	Negative	N/A	N/A	No	N/A	N/A		This study
70	*Coronella austriaca*	2022-08-07	41.6	13.1	Lazio	Juvenile	No	Dry Swab (3)	Negative	N/A	N/A	No	N/A	N/A		This study
71	*Hierophis viridiflavus* sspp.	2022-08-10	44.1	11.9	Emilia-Romagna	Adult	No	Dry Swab (3)	Negative	N/A	N/A	No	N/A	N/A		This study
72	*Zamenis longissimus*	2022-06-12	43.9	11.8	Emilia-Romagna	Adult	No	Dry Swab (3)	Negative	N/A	N/A	No	N/A	N/A		This study
73	*Coronella austriaca*	2022-08-16	39.9	16.2	Basilicata	Juvenile	No	Dry Swab (3)	Negative	N/A	N/A	No	N/A	N/A		This study
74	*Vipera berus marasso*	2022-07-02	46.0	9.6	Lombardy	Adult	No	Dry Swab (3)	Negative	N/A	N/A	No	N/A	N/A		This study
75	*Vipera berus marasso*	2022-07-03	46.0	9.6	Lombardy	Adult	No	Dry Swab (3)	Negative	N/A	N/A	No	N/A	N/A		This study
76	*Natrix helvetica sicula*	2022-08-10	42.2	13.9	Abruzzo	Juvenile	No	Dry Swab (3)	Negative	N/A	N/A	No	N/A	N/A		This study
77	*Vipera ursinii*	2022-08-19	42.4	13.6	Abruzzo	Adult	No	Dry Swab (3)	Negative	N/A	N/A	No	N/A	N/A		This study
78	*Vipera ursinii*	2022-08-19	42.4	13.6	Abruzzo	Adult	No	Dry Swab (3)	Negative	N/A	N/A	No	N/A	N/A		This study
79	*Coronella austriaca*	2022-07-04	46.1	9.6	Lombardy	Adult	No	Dry Swab (3)	Negative	N/A	N/A	No	N/A	N/A		This study
80	*Natrix maura*	2022 May	39.4	9.5	Sardinia	Subadult	No	Dry Swab (3)	Negative	N/A	N/A	No	N/A	N/A		This study
81	*Natrix maura*	2022 May	39.4	9.5	Sardinia	Subadult	No	Dry Swab (3)	Negative	N/A	N/A	No	N/A	N/A		This study

82	*Hierophis viridiflavus* sspp.	2022 May	39.4	9.5	Sardinia	Subadult	No	Dry Swab (3)	Negative	N/A	N/A	No	N/A	N/A		This study
83	*Natrix helvetica sicula*	2022-07-28	37.9	14.7	Sicily	Adult	N/A	Molt (frozen)	Negative	N/A	N/A	No	N/A	N/A	Shed	This study
84	*Coronella austriaca*	2022-05-21	38.1	14.8	Sicily	Adult	N/A	Molt (frozen)	Negative	N/A	N/A	No	N/A	N/A	Shed	This study
85	*Natrix helvetica sicula*	2022-07-12	37.1	15.0	Sicily	Adult	No	Dry Swab (3)	Negative	N/A	N/A	No	N/A	N/A		This study
86	*Natrix helvetica sicula*	N/A	38.0	13.0	Sicily	Adult	No	Dry Swab (3)	Negative	N/A	N/A	No	N/A	N/A		This study
87	*Coronella austriaca*	2022-10-14	36.9	15.0	Sicily	Adult	No	Dry Swab (3)	Negative	N/A	N/A	No	N/A	N/A		This study
88	*Hierophis viridiflavus* sspp.	2022-10-16	37.7	14.9	Sicily	Adult	N/A	Molt (frozen)	Negative	N/A	N/A	No	N/A	N/A	Shed	This study
89	*Natrix helvetica sicula*	2022-10-14	37.1	15.0	Sicily	Adult	No	Dry Swab (3)	Negative	N/A	N/A	No	N/A	N/A		This study
90	*Hierophis viridiflavus* sspp.	2022 June	37.1	15.0	Sicily	Adult	N/A	Molt (frozen)	Negative	N/A	N/A	No	N/A	N/A	Shed	This study
91	*Coronella austriaca*	2022 June	37.8	14.9	Sicily	Adult	No	Dry Swab (3)	Negative	N/A	N/A	No	N/A	N/A		This study
92	*Hierophis viridiflavus* sspp.	2022-10-14	37.1	15.0	Sicily	Adult	N/A	Molt (frozen)	Negative	N/A	N/A	No	N/A	N/A	Shed	This study
93	*Natrix helvetica sicula*	2022-10-05	36.9	15.0	Sicily	Adult	No	Tissue (in alcohol)	Negative	N/A	N/A	No	N/A	N/A	Found dead	This study
94	*Vipera aspis* sspp.	2022-10-14	37.1	15.0	Sicily	Juvenile	No	Dry Swab (3)	Negative	N/A	N/A	No	N/A	N/A		This study
95	*Zamenis longissimus*	2022-07-07	44.5	9.1	Liguria	Adult	No	Dry Swab (3) + Tissue (frozen)	Negative	N/A	N/A	No	N/A	N/A	Found dead	This study
96	*Hierophis viridiflavus* sspp.	2022-07-06	45.1	9.6	Emilia-Romagna	Juvenile	No	Dry Swab (3)	Negative	N/A	N/A	No	N/A	N/A		This study
97	*Vipera aspis* sspp.	2022-08-08	41.7	13.9	Lazio	Adult	N/A	Molt (frozen)	Negative	N/A	N/A	No	N/A	N/A	Shed	This study
98	*Elaphe quatuorlineata*	2022 May	42.0	13.8	Abruzzo	Adult	N/A	Molt (frozen)	Negative	N/A	N/A	No	N/A	N/A	Shed	This study
99	*Elaphe quatuorlineata*	2022 May	42.0	13.8	Abruzzo	Adult	N/A	Molt (frozen)	Negative	N/A	N/A	No	N/A	N/A	Shed	This study
100	*Hierophis viridiflavus* sspp.	2022-07-20	39.5	9.4	Sardinia	Adult	No	Dry Swab (3)	Negative	N/A	N/A	No	N/A	N/A		This study
101	*Hierophis viridiflavus* sspp.	2022-07-21	39.7	9.4	Sardinia	Adult	No	Dry Swab (3)	Negative	N/A	N/A	No	N/A	N/A		This study
102	*Natrix maura*	2022-07-21	40.0	9.4	Sardinia	Subadult	No	Dry Swab (3)	Negative	N/A	N/A	No	N/A	N/A		This study

103	*Natrix maura*	2022-07-25	39.9	9.3	Sardinia	Subadult	No	Dry Swab (3)	Negative	N/A	N/A	No	N/A	N/A		This study
104	*Natrix maura*	2022-07-22	39.4	9.5	Sardinia	Subadult	No	Dry Swab (3)	Negative	N/A	N/A	No	N/A	N/A		This study
105	*Natrix maura*	2022-07-22	39.3	9.2	Sardinia	Subadult	No	Dry Swab (3)	Negative	N/A	N/A	No	N/A	N/A		This study
106	*Natrix maura*	2022-07-22	39.3	9.2	Sardinia	Subadult	No	Dry Swab (3)	Negative	N/A	N/A	No	N/A	N/A		This study
107	*Natrix maura*	2022-07-22	39.3	9.2	Sardinia	Subadult	No	Dry Swab (3)	Negative	N/A	N/A	No	N/A	N/A		This study
108	*Hierophis viridiflavus* sspp.	2022-07-21	39.4	9.2	Sardinia	Adult	N/A	Molt (frozen)	Negative	N/A	N/A	No	N/A	N/A	Shed	This study
109	*Hierophis viridiflavus* sspp.	2022-09-26	37.9	15.0	Sicily	Adult	N/A	Molt (frozen)	Negative	N/A	N/A	No	N/A	N/A	Shed	This study
110	*Hierophis viridiflavus* sspp.	2022 June	37.1	15.0	Sicily	Adult	N/A	Molt (frozen)	Negative	N/A	N/A	No	N/A	N/A	Shed	This study
111	*Hierophis viridiflavus* sspp.	2022-07-12	37.1	15.0	Sicily	Juvenile	N/A	Molt (frozen)	Negative	N/A	N/A	No	N/A	N/A	Shed	This study
112	*Hierophis viridiflavus* sspp.	2022-09-06	38.0	12.1	Sicily	Adult	N/A	Molt (frozen)	Negative	N/A	N/A	No	N/A	N/A	Shed	This study
113	*Hierophis viridiflavus* sspp.	2022-09-26	37.9	14.9	Sicily	Adult	N/A	Molt (frozen)	Negative	N/A	N/A	No	N/A	N/A	Shed	This study
114	*Hierophis viridiflavus* sspp.	2022-05-01	42.2	14.1	Abruzzo	Adult	No	Dry Swab (3)	Negative	N/A	N/A	No	N/A	N/A	Pretoro Serpari fest	This study
115	*Hierophis viridiflavus* sspp.	2022-05-01	42.2	14.1	Abruzzo	Adult	No	Dry Swab (3)	Negative	N/A	N/A	No	N/A	N/A	Pretoro Serpari fest	This study
116	*Hierophis viridiflavus* sspp.	2022-05-01	42.2	14.1	Abruzzo	Adult	No	Dry Swab (3)	Negative	N/A	N/A	No	N/A	N/A	Pretoro Serpari fest	This study
117	*Hierophis viridiflavus* sspp.	2022-05-01	42.2	14.1	Abruzzo	Adult	No	Dry Swab (3)	Negative	N/A	N/A	No	N/A	N/A	Pretoro Serpari fest	This study
118	*Hierophis viridiflavus* sspp.	2022-05-01	42.2	14.1	Abruzzo	Adult	No	Dry Swab (3)	Negative	N/A	N/A	No	N/A	N/A	Pretoro Serpari fest	This study
119	*Hierophis viridiflavus* sspp.	2022-05-01	42.2	14.1	Abruzzo	Adult	No	Dry Swab (3)	Negative	N/A	N/A	No	N/A	N/A	Pretoro Serpari fest	This study
120	*Hierophis viridiflavus* sspp.	2022-05-01	42.2	14.1	Abruzzo	Adult	No	Dry Swab (3)	Negative	N/A	N/A	No	N/A	N/A	Pretoro Serpari fest	This study
121	*Hierophis viridiflavus* sspp.	2022-05-01	42.2	14.1	Abruzzo	Adult	No	Dry Swab (3)	Negative	N/A	N/A	No	N/A	N/A	Pretoro Serpari fest	This study
122	*Hierophis viridiflavus* sspp.	2022-05-01	42.2	14.1	Abruzzo	Adult	No	Dry Swab (3)	Negative	N/A	N/A	No	N/A	N/A	Pretoro Serpari fest	This study
123	*Zamenis longissimus*	2022-05-01	42.2	14.1	Abruzzo	Adult	No	Dry Swab (3)	Negative	N/A	N/A	No	N/A	N/A	Pretoro Serpari fest	This study

124	*Natrix helvetica sicula*	2022-05-01	42.2	14.1	Abruzzo	Adult	Yes	Dry Swab (3)	Negative	N/A	N/A	No	N/A	N/A	Pretoro Serpari fest	This study
125	*Natrix helvetica sicula*	2022-05-01	42.2	14.1	Abruzzo	Adult	No	Dry Swab (3)	Negative	N/A	N/A	No	N/A	N/A	Pretoro Serpari fest	This study
126	*Natrix tessellata*	2022-04-18	45.8	9.4	Lombardy	Juvenile	No	Dry Swab (3)	Negative	N/A	N/A	No	N/A	N/A		This study
127	*Elaphe quatuorlineata*	2022 April	42.0	13.8	Abruzzo	Adult	No	Dry Swab (3)	Negative	N/A	N/A	No	N/A	N/A	Cocullo Serpari fest	This study
128	*Elaphe quatuorlineata*	2022 April	42.0	13.8	Abruzzo	Adult	No	Dry Swab (3)	Negative	N/A	N/A	No	N/A	N/A	Cocullo Serpari fest	This study
129	*Elaphe quatuorlineata*	2022 April	42.0	13.8	Abruzzo	Adult	No	Dry Swab (3)	Negative	N/A	N/A	No	N/A	N/A		This study
130	*Elaphe quatuorlineata*	2022 April	42.0	13.8	Abruzzo	Adult	No	Dry Swab (3)	Negative	N/A	N/A	No	N/A	N/A		This study
131	*Elaphe quatuorlineata*	2022 April	42.0	13.8	Abruzzo	Adult	No	Dry Swab (3)	Negative	N/A	N/A	No	N/A	N/A	Cocullo Serpari fest	This study
132	*Macroprotodon* cf. *cucullatus*	2022-04-15	35.5	12.6	Sicily	Adult	N/A	Molt (frozen)	Negative	N/A	N/A	No	N/A	N/A	Shed	This study
133	*Malpolon insignitus*	2022-04-15	35.5	12.6	Sicily	Adult	No	Dry Swab (3) + Tissue (frozen)	Negative	N/A	N/A	No	N/A	N/A		This study
134	*Malpolon insignitus*	2022-04-15	35.5	12.6	Sicily	Adult	N/A	Molt (frozen)	Negative	N/A	N/A	No	N/A	N/A	Shed	This study
135	*Macroprotodon* cf. *cucullatus*	2022-04-16	35.5	12.6	Sicily	Adult	N/A	Molt (frozen)	Negative	N/A	N/A	No	N/A	N/A	Shed	This study
136	*Malpolon insignitus*	2022-04-14	35.5	12.6	Sicily	Adult	N/A	Molt (frozen)	Negative	N/A	N/A	No	N/A	N/A	Shed	This study
137	*Macroprotodon* cf. *cucullatus*	2022-04-14	35.5	12.5	Sicily	Adult	No	Dry Swab (3)	Negative	N/A	N/A	No	N/A	N/A		This study
138	*Macroprotodon* cf. *cucullatus*	2022-04-14	35.5	12.6	Sicily	Juvenile	No	Dry Swab (3)	Negative	N/A	N/A	No	N/A	N/A		This study
139	*Macroprotodon* cf. *cucullatus*	2022-04-14	35.5	12.6	Sicily	Adult	No	Dry Swab (3)	Negative	N/A	N/A	No	N/A	N/A		This study
140	*Macroprotodon* cf. *cucullatus*	2022-04-14	35.5	12.5	Sicily	Adult	No	Dry Swab (3)	Negative	N/A	N/A	No	N/A	N/A		This study
141	*Macroprotodon* cf. *cucullatus*	2022-04-14	35.5	12.5	Sicily	Adult	No	Dry Swab (3)	Negative	N/A	N/A	No	N/A	N/A		This study
142	*Macroprotodon* cf. *cucullatus*	2022-04-13	35.5	12.6	Sicily	Adult	N/A	Molt (frozen)	Negative	N/A	N/A	No	N/A	N/A	Shed	This study
143	*Macroprotodon* cf. *cucullatus*	2022-04-13	35.5	12.6	Sicily	Adult	N/A	Molt (frozen)	Negative	N/A	N/A	No	N/A	N/A	Shed	This study
144	*Macroprotodon* cf. *cucullatus*	2022-04-13	35.5	12.6	Sicily	Adult	N/A	Molt (frozen)	Negative	N/A	N/A	No	N/A	N/A	Shed	This study

145	*Macroprotodon* cf. *cucullatus*	2022 April	35.5	12.6	Sicily	Adult	No	Dry Swab (3)	Negative	N/A	N/A	No	N/A	N/A		This study
146	*Macroprotodon* cf. *cucullatus*	2022 April	35.5	12.6	Sicily	Adult	No	Dry Swab (3)	Negative	N/A	N/A	No	N/A	N/A		This study
147	*Macroprotodon* cf. *cucullatus*	2022 April	35.5	12.5	Sicily	Adult	No	Dry Swab (3)	Negative	N/A	N/A	No	N/A	N/A		This study
148	*Macroprotodon* cf. *cucullatus*	2022 April	35.5	12.6	Sicily	Adult	No	Dry Swab (3)	Negative	N/A	N/A	No	N/A	N/A		This study
149	*Macroprotodon* cf. *cucullatus*	2022 April	35.5	12.6	Sicily	Adult	N/A	Molt (frozen)	Negative	N/A	N/A	No	N/A	N/A	Shed	This study
150	*Hierophis viridiflavus* sspp.	2022-05-27	44.4	9.3	Liguria	Adult	No	Dry Swab (3)	Negative	N/A	N/A	No	N/A	N/A		This study
151	*Natrix tessellata*	2022-06-03	44.1	9.9	Liguria	Adult	No	Dry Swab (3)	Negative	N/A	N/A	No	N/A	N/A	In stream, underwater	This study
152	*Hierophis viridiflavus* sspp.	2022-06-04	45.7	8.7	Lombardy	Adult	No	Dry Swab (3)	Negative	N/A	N/A	No	N/A	N/A		This study
153	*Natrix helvetica sicula*	2021-09-10	37.9	13.4	Sicily	Adult	N/A	Molt (frozen)	Negative	N/A	N/A	No	N/A	N/A	Shed	This study
154	*Natrix maura*	2022-05-18	39.3	9.4	Sardinia	Juvenile	No	Dry Swab (3)	Negative	N/A	N/A	No	N/A	N/A		This study
155	*Natrix maura*	2022-05-18	39.3	9.4	Sardinia	Juvenile	No	Dry Swab (3)	Negative	N/A	N/A	No	N/A	N/A		This study
156	*Natrix maura*	2022-05-19	39.2	9.1	Sardinia	Adult	No	Dry Swab (3)	Negative	N/A	N/A	No	N/A	N/A		This study
157	*Natrix helvetica cetti*	2022-05-18	39.3	9.4	Sardinia	Adult	No	Dry Swab (3)	Negative	N/A	N/A	No	N/A	N/A		This study
158	*Natrix helvetica cetti*	2022-05-18	39.3	9.4	Sardinia	Adult	No	Dry Swab (3)	Negative	N/A	N/A	No	N/A	N/A		This study
159	*Hierophis viridiflavus* sspp.	2022-05-18	39.3	9.4	Sardinia	Adult	No	Dry Swab (3)	Negative	N/A	N/A	No	N/A	N/A		This study
160	*Hierophis viridiflavus* sspp.	2022-05-18	39.3	9.4	Sardinia	Adult	No	Dry Swab (3)	Negative	N/A	N/A	No	N/A	N/A	Found dead	This study
161	*Malpolon monspessulanus*	2016-05-20	44.1	8.2	Liguria	Adult	N/A	Tissue (frozen)	Negative	N/A	N/A	No	N/A	N/A	Found dead	This study
162	*Malpolon monspessulanus*	2020 April	44.1	8.2	Liguria	Adult	N/A	Tissue (frozen)	Negative	N/A	N/A	No	N/A	N/A	Found dead	This study
163	*Malpolon monspessulanus*	2020 May	44.4	8.5	Liguria	Adult	N/A	Tissue (frozen)	Negative	N/A	N/A	No	N/A	N/A	Found dead	This study
164	*Malpolon monspessulanus*	2021 May	44.3	8.5	Liguria	Adult	N/A	Tissue (frozen)	Negative	N/A	N/A	No	N/A	N/A	Found dead	This study
165	*Coronella girondica*	N/A	44.4	8.5	Liguria	Adult	N/A	Tissue (frozen)	Negative	N/A	N/A	No	N/A	N/A	Found dead	This study

166	*Malpolon monspessulanus*	N/A	44.6	10.9	Emilia-Romagna	Adult	N/A	Tissue (frozen)	Negative	N/A	N/A	No	N/A	N/A	Moved individual	This study
167	*Zamenis longissimus*	2021-08-25	44.4	8.5	Liguria	Adult	N/A	Tissue (frozen)	Negative	N/A	N/A	No	N/A	N/A	Found dead	This study
168	*Zamenis longissimus*	2020-04-08	44.1	8.2	Liguria	Adult	N/A	Tissue (frozen)	Negative	N/A	N/A	No	N/A	N/A	Found dead	This study
169	*Zamenis longissimus*	N/A	44.1	8.2	Liguria	Adult	N/A	Tissue (frozen)	Negative	N/A	N/A	No	N/A	N/A	Found dead	This study
170	*Malpolon monspessulanus*	N/A	44.1	8.2	Liguria	Adult	N/A	Tissue (frozen)	Negative	N/A	N/A	No	N/A	N/A	Found dead	This study
171	*Malpolon monspessulanus*	N/A	44.1	8.2	Liguria	Adult	N/A	Tissue (frozen)	Negative	N/A	N/A	No	N/A	N/A	Found dead	This study
172	*Hierophis viridiflavus* sspp.	N/A	N/A	N/A	Liguria	Adult	N/A	Tissue (frozen)	Negative	N/A	N/A	No	N/A	N/A	Found dead	This study
173	*Coronella girondica*	N/A	44.1	8.2	Liguria	Adult	N/A	Tissue (frozen)	Negative	N/A	N/A	No	N/A	N/A	Found dead	This study
174	*Vipera aspis* sspp.	2022-03-24	45.8	13.1	Friuli-Venezia Giulia	Adult	No	Dry Swab (3)	Negative	N/A	N/A	No	N/A	N/A		This study
175	*Vipera aspis* sspp.	2022-03-24	45.8	13.1	Friuli-Venezia Giulia	Adult	No	Dry Swab (3)	Negative	N/A	N/A	No	N/A	N/A		This study
176	*Vipera ammodytes*	2022-03-25	46.2	13.0	Friuli-Venezia Giulia	Adult	No	Dry Swab (3)	Negative	N/A	N/A	No	N/A	N/A		This study
177	*Natrix helvetica cetti*	2022-03-21	39.4	8.6	Sardinia	Adult	No	Dry Swab (3)	Negative	N/A	N/A	No	N/A	N/A		This study
178	*Hierophis viridiflavus* sspp.	2022-05-10	45.7	9.4	Lombardy	Adult	N/A	Molt (frozen)	Negative	N/A	N/A	No	N/A	N/A	Shed	This study
179	*Hierophis viridiflavus* sspp.	2022 April	38.0	13.3	Sicily	Adult	N/A	Molt (frozen)	Negative	N/A	N/A	No	N/A	N/A	Shed	This study
180	*Vipera aspis* sspp.	2021-08-08	41.7	13.9	Lazio	Adult	N/A	Molt (frozen)	Negative	N/A	N/A	No	N/A	N/A	Shed	This study
181	*Vipera aspis* sspp.	2021-08-08	41.7	13.9	Lazio	Adult	N/A	Molt (frozen)	Negative	N/A	N/A	No	N/A	N/A	Shed	This study
182	*Hierophis viridiflavus* sspp.	2022-04-16	43.9	8.0	Liguria	Adult	N/A	Molt (frozen)	Negative	N/A	N/A	No	N/A	N/A	Shed	This study
183	*Coronella austriaca*	2021 May	45.7	7.9	Piedmont	Adult	N/A	Molt (frozen)	Negative	N/A	N/A	No	N/A	N/A	Shed	This study
184	*Hierophis viridiflavus* sspp.	2022-02-05	45.7	9.5	Lombardy	Adult	N/A	Molt (frozen)	Negative	N/A	N/A	No	N/A	N/A	Shed	This study
185	*Vipera aspis* sspp.	2022-10-04	N/A	N/A	Sicily	Adult	N/A	Molt (frozen)	Negative	N/A	N/A	No	N/A	N/A	Shed	This study
186	*Vipera aspis* sspp.	2021-10-17	37.7	14.9	Sicily	Adult	No	Dry Swab (1)	Negative	N/A	N/A	No	N/A	N/A		This study

187	*Hierophis viridiflavus* sspp.	2015-06-01	44.3	8.5	Liguria	Adult	No	Dry Swab (1)	Negative	N/A	N/A	No	N/A	N/A		This study
188	*Natrix tessellata*	2021-02-28	45.9	10.9	Trentino-South Tyrol	Adult	No	Dry Swab (1)	Positive	31	II	No	N/A	Oo present		This study
189	*Natrix tessellata*	2021-02-28	45.9	10.8	Trentino-South Tyrol	Juvenile	No	Dry Swab (2)	Negative	N/A	N/A	No	N/A	N/A		This study
190	*Natrix tessellata*	2021-03-09	45.9	10.9	Trentino-South Tyrol	Juvenile	No	Dry Swab (2)	Negative	N/A	N/A	No	N/A	N/A		This study
191	*Natrix tessellata*	2021-03-13	45.9	10.8	Trentino-South Tyrol	Adult	No	Dry Swab (2) + Tissue (frozen)	Positive	88	N/A	No	N/A	Oo present		This study
192	*Natrix tessellata*	2021-03-14	45.9	10.9	Trentino-South Tyrol	Juvenile	Yes	Dry Swab (2)	Positive	29	N/A	No	N/A	Apparent Ophidiomycosis		This study
193	*Natrix tessellata*	2021-03-14	45.9	10.8	Trentino-South Tyrol	Adult	No	Dry Swab (2) + Tissue (frozen)	Positive	13	II	No	N/A	Oo present		This study
194	*Natrix tessellata*	2021-03-14	45.9	10.8	Trentino-South Tyrol	Adult	Yes	Dry Swab (2) + Tissue (frozen)	Positive	72	N/A	No	N/A	Apparent Ophidiomycosis		This study
195	*Natrix tessellata*	2021-03-15	45.9	10.8	Trentino-South Tyrol	Juvenile	Yes	Dry Swab (2) + Tissue (frozen)	Positive	83	N/A	No	N/A	Apparent Ophidiomycosis		This study
196	*Natrix tessellata*	2021-03-24	45.9	10.9	Trentino-South Tyrol	Adult	No	Dry Swab (2) + Tissue (frozen)	Positive	28	II	No	N/A	Oo present		This study
197	*Natrix tessellata*	2021-03-28	45.9	10.8	Trentino-South Tyrol	Juvenile	No	Dry Swab (2)	Negative	N/A	N/A	No	N/A	N/A		This study
198	*Natrix tessellata*	2021-03-28	45.9	10.8	Trentino-South Tyrol	Juvenile	No	Dry Swab (2)	Negative	N/A	N/A	No	N/A	N/A		This study
199	*Natrix tessellata*	2021-03-28	45.9	10.8	Trentino-South Tyrol	Adult	Yes	Dry Swab (2)	Positive	52	II	No	N/A	Apparent Ophidiomycosis		This study
200	*Natrix tessellata*	2021-03-28	45.9	10.8	Trentino-South Tyrol	Juvenile	No	Dry Swab (2)	Negative	N/A	N/A	No	N/A	N/A		This study
201	*Natrix tessellata*	2021-03-28	45.9	10.8	Trentino-South Tyrol	Juvenile	Yes	Dry Swab (2)	Negative	N/A	N/A	No	N/A	N/A		This study
202	*Natrix tessellata*	2021-04-01	45.9	10.8	Trentino-South Tyrol	Adult	No	Dry Swab (2)	Negative	N/A	N/A	No	N/A	N/A		This study
203	*Natrix tessellata*	2021-04-01	45.9	10.9	Trentino-South Tyrol	Adult	No	Dry Swab (2)	Negative	N/A	N/A	No	N/A	N/A		This study
204	*Natrix tessellata*	2021-04-01	45.9	10.9	Trentino-South Tyrol	Adult	No	Dry Swab (2)	Negative	N/A	N/A	No	N/A	N/A		This study
205	*Natrix tessellata*	2021-04-01	45.9	10.9	Trentino-South Tyrol	Adult	No	Dry Swab (2)	Negative	N/A	N/A	No	N/A	N/A		This study
206	*Natrix tessellata*	2021-04-01	45.9	10.9	Trentino-South Tyrol	Adult	No	Dry Swab (2)	Negative	N/A	N/A	No	N/A	N/A		This study
207	*Natrix tessellata*	2021-04-01	45.9	10.9	Trentino-South Tyrol	Adult	No	Dry Swab (2)	Negative	N/A	N/A	No	N/A	N/A		This study

208	*Natrix tessellata*	2021-04-01	45.9	10.9	Trentino-South Tyrol	Adult	No	Dry Swab (2)	Negative	N/A	N/A	No	N/A	N/A		This study
209	*Natrix tessellata*	2021-04-01	45.9	10.9	Trentino-South Tyrol	Adult	No	Dry Swab (2)	Negative	N/A	N/A	No	N/A	N/A		This study
210	*Natrix tessellata*	2023-05-21	45.9	8.5	Piedmont	Subadult	Yes	Dry Swab (3) + Tissue (in formalin)	Positive	42	II (IIDE)	Yes	Negative (inflammation)	Apparent Ophidiomycosis		This study
211	*Natrix tessellata*	2023-05-21	45.9	8.5	Piedmont	Subadult	Yes	Dry Swab (3) + Tissue (in formalin)	Positive	32	II (IIDE)	Yes	Negative (inflammation)	Apparent Ophidiomycosis		This study
212	*Natrix tessellata*	2023-05-21	45.9	8.5	Piedmont	Subadult	Yes	Dry Swab (3) + Tissue (in formalin)	Negative	N/A	N/A	No	N/A	N/A		This study
213	*Natrix helvetica sicula*	2023-05-27	45.9	8.5	Piedmont	Subadult	No	Dry Swab (2)	Negative	N/A	N/A	No	N/A	N/A		This study
214	*Natrix tessellata*	2023-06-03	45.7	8.6	Lombardy	Adult	No	Dry Swab (2)	Negative	N/A	N/A	No	N/A	N/A		This study
215	*Natrix tessellata*	2023-06-16	46.1	9.3	Lombardy	Adult	No	Dry Swab (2)	Negative	N/A	N/A	No	N/A	N/A		This study
216	*Natrix tessellata*	2023-06-16	46.1	9.3	Lombardy	Adult	No	Dry Swab (2)	Negative	N/A	N/A	No	N/A	N/A		This study
217	*Natrix tessellata*	2023-06-16	46.1	9.3	Lombardy	Adult	Yes	Dry Swab (3) + Tissue (in formalin)	Negative	N/A	N/A	No	N/A	N/A		This study
218	*Natrix tessellata*	2023-06-16	46.1	9.3	Lombardy	Adult	Yes	Dry Swab (3) + Tissue (in formalin)	Negative	N/A	N/A	No	N/A	N/A		This study
219	*Natrix tessellata*	2023-06-16	46.1	9.3	Lombardy	Subadult	No	Dry Swab (2)	Positive	16	N/A	No	N/A	Oo present		This study
220	*Natrix tessellata*	2023-06-16	46.1	9.3	Lombardy	Subadult	No	Dry Swab (2)	Negative	N/A	N/A	No	N/A	N/A		This study
221	*Natrix tessellata*	2023-07-07	46.1	9.3	Lombardy	Subadult	No	Dry Swab (2)	Negative	N/A	N/A	No	N/A	N/A		This study
222	*Natrix tessellata*	2023-07-07	46.1	9.3	Lombardy	Adult	No	Dry Swab (2)	Negative	N/A	N/A	No	N/A	N/A		This study
223	*Natrix tessellata*	2023-07-07	46.1	9.3	Lombardy	Adult	No	Dry Swab (2)	Negative	N/A	N/A	No	N/A	N/A		This study
224	*Natrix tessellata*	2023-07-07	46.1	9.3	Lombardy	Adult	No	Dry Swab (2)	Negative	N/A	N/A	No	N/A	N/A		This study
225	*Natrix tessellata*	2023-08-02	46.1	9.3	Lombardy	Adult	No	Dry Swab (2)	Positive	31	II	No	N/A	Oo present		This study
226	*Natrix helvetica sicula*	2023-08-18	45.9	8.5	Piedmont	Subadult	No	Dry Swab (2)	Negative	N/A	N/A	No	N/A	N/A		This study
227	*Natrix tessellata*	2023-08-30	46.1	9.3	Lombardy	Subadult	No	Dry Swab (3)	Negative	N/A	N/A	No	N/A	N/A		This study
228	*Natrix tessellata*	2023-08-30	46.1	9.3	Lombardy	Juvenile	No	Dry Swab (3)	Positive	314	II (IIDE)	No	N/A	Oo present		This study

229	*Natrix tessellata*	2023-08-30	46.1	9.3	Lombardy	Juvenile	No	Dry Swab (3)	Positive	70	II (IIDE)	No	N/A	Oo present		This study
230	*Natrix tessellata*	2023-08-30	46.1	9.3	Lombardy	Adult	No	Dry Swab (3)	Negative	N/A	N/A	No	N/A	N/A		This study
231	*Natrix tessellata*	2023-08-30	46.1	9.3	Lombardy	Adult	Yes	Dry Swab (3) + Tissue (in formalin)	Negative	N/A	N/A	No	N/A	N/A		This study
232	*Natrix tessellata*	2023-09-02	45.9	10.8	Trentino-South Tyrol	Adult	Yes	Dry Swab (3) + Tissue (in formalin)	Negative	N/A	N/A	No	N/A	N/A		This study
233	*Hierophis viridiflavus* sspp.	2023-03-22	45.2	9.2	Lombardy	Adult	No	Dry Swab (2) + Tissue (in formalin)	Negative	N/A	N/A	No	N/A	N/A		This study
234	*Hierophis viridiflavus* sspp.	2023-03-22	45.2	9.2	Lombardy	Adult	Yes	Dry Swab (2) + Tissue(in formalin)	Negative	N/A	N/A	No	N/A	N/A		This study
235	*Hierophis viridiflavus* sspp.	2023-03-22	45.2	9.2	Lombardy	Adult	No	Dry Swab (2) + Tissue (in formalin)	Negative	N/A	N/A	No	N/A	N/A		This study
236	*Hierophis viridiflavus* sspp.	2023-03-23	45.2	9.2	Lombardy	Adult	No	Dry Swab (2)	Negative	N/A	N/A	No	N/A	N/A		This study
237	*Hierophis viridiflavus* sspp.	2023-03-23	45.2	9.2	Lombardy	Adult	No	Dry Swab (2) + Tissue (in formalin)	Negative	N/A	N/A	No	N/A	N/A		This study
238	*Hierophis viridiflavus* sspp.	2023-03-23	45.2	9.2	Lombardy	Adult	No	Dry Swab (2)	Negative	N/A	N/A	No	N/A	N/A		This study
239	*Hierophis viridiflavus* sspp.	2023-03-23	45.2	9.2	Lombardy	Adult	No	Dry Swab (2)	Negative	N/A	N/A	No	N/A	N/A		This study
240	*Coronella girondica*	2023-04-14	44.9	9.2	Lombardy	Adult	No	Dry Swab (3)	Negative	N/A	N/A	No	N/A	N/A		This study
241	*Natrix maura*	2023-04-16	44.9	9.0	Lombardy	Adult	No	Dry Swab (3)	Negative	N/A	N/A	No	N/A	N/A		This study
242	*Coronella girondica*	2023-04-26	44.9	9.3	Lombardy	Adult	No	Dry Swab (3)	Negative	N/A	N/A	No	N/A	N/A		This study
243	*Hierophis viridiflavus* sspp.	2023-05-03	46.0	8.4	Piedmont	Adult	No	Dry Swab (3) + Tissue (in formalin)	Positive	66	II (IIDE)	Yes	Hyphae and arthroconidia	Ophidiomycosis and Oo shedder		This study
244	*Natrix helvetica sicula*	2023-05-04	46.0	8.4	Piedmont	Subadult	N/A	Dry Swab (3)	Negative	N/A	N/A	No	N/A	N/A	Found dead	This study
245	*Vipera aspis* sspp.	2023-05-15	45.9	8.2	Piedmont	Adult	No	Dry Swab (3)	Negative	N/A	N/A	No	N/A	N/A		This study
246	*Hierophis viridiflavus* sspp.	2023-05-15	46.0	8.4	Piedmont	Adult	N/A	Dry Swab (3)	Negative	N/A	N/A	No	N/A	N/A	Found dead	This study
247	*Vipera aspis* sspp.	2023-05-16	46.0	8.4	Piedmont	Adult	No	Dry Swab (3)	Negative	N/A	N/A	No	N/A	N/A		This study
248	*Hierophis viridiflavus* sspp.	2023-05-22	45.8	13.5	Friuli-Venezia Giulia	Adult	N/A	Dry Swab (3)	Negative	N/A	N/A	No	N/A	N/A	Found dead	This study

249	*Hierophis viridiflavus* sspp.	2023-05-25	45.8	13.5	Friuli-Venezia Giulia	Adult	No	Dry Swab (3) + Tissue (in formalin)	Negative	N/A	N/A	No	N/A	N/A		This study
250	*Hierophis viridiflavus* sspp.	2023-05-22	45.4	10.6	Lombardy	Adult	Yes	Dry Swab (3)	Negative	N/A	N/A	No	N/A	N/A		This study
251	*Hierophis viridiflavus* sspp.	2023-05-22	45.4	10.6	Lombardy	Adult	No	Dry Swab (3)	Negative	N/A	N/A	No	N/A	N/A		This study
252	*Hierophis viridiflavus* sspp.	2023-05-23	45.1	10.9	Lombardy	Adult	No	Dry Swab (3)	Negative	N/A	N/A	No	N/A	N/A		This study
253	*Natrix tessellata*	2023-05-25	45.2	10.7	Lombardy	Adult	No	Dry Swab (3)	Negative	N/A	N/A	No	N/A	N/A		This study
254	*Natrix helvetica sicula*	2023-05-30	45.9	8.2	Piedmont	Juvenile	No	Dry Swab (3)	Negative	N/A	N/A	No	N/A	N/A		This study
255	*Natrix helvetica sicula*	2023-05-30	45.9	8.2	Piedmont	Juvenile	No	Dry Swab (3)	Negative	N/A	N/A	No	N/A	N/A		This study
256	*Vipera aspis* sspp.	2023-05-30	45.9	8.2	Piedmont	Adult	No	Dry Swab (3)	Negative	N/A	N/A	No	N/A	N/A		This study
257	*Zamenis longissimus*	2023-05-30	45.9	8.3	Piedmont	Adult	N/A	Dry Swab (3)	Negative	N/A	N/A	No	N/A	N/A	Found dead	This study
258	*Hierophis viridiflavus* sspp.	2023-05-31	46.0	8.4	Piedmont	Adult	Yes	Dry Swab (2) + Tissue (in formalin)	Positive	619	II (IIDE)	Yes	Hyphae and arthroconidia	Ophidiomycosis and Oo shedder		This study
259	*Hierophis viridiflavus* sspp.	2023-05-28	45.2	9.5	Lombardy	Adult	N/A	Dry Swab (3)	Negative	N/A	N/A	No	N/A	N/A	Found dead	This study
260	*Hierophis viridiflavus* sspp.	2023-05-31	45.2	9.5	Lombardy	Adult	N/A	Dry Swab (3)	Negative	N/A	N/A	No	N/A	N/A	Found dead	This study
261	*Hierophis viridiflavus* sspp.	2022-05-28	45.2	9.9	Lombardy	Adult	N/A	Dry Swab (3)	Negative	N/A	N/A	No	N/A	N/A	Found dead	This study
262	*Hierophis viridiflavus* sspp.	2022-07-20	45.2	9.1	Lombardy	Adult	N/A	Dry Swab (3)	Negative	N/A	N/A	No	N/A	N/A	Found dead	This study
263	*Hierophis viridiflavus* sspp.	2022-05-18	45.2	9.0	Lombardy	Adult	N/A	Dry Swab (3)	Negative	N/A	N/A	No	N/A	N/A	Found dead	This study
264	*Hierophis viridiflavus* sspp.	2023-05-31	45.2	8.8	Lombardy	N/A	N/A	Dry Swab (3)	Negative	N/A	N/A	No	N/A	N/A	Found dead	This study
265	*Hierophis viridiflavus* sspp.	2023-06-02	44.7	8.7	Piedmont	Adult	N/A	Dry Swab (3)	Negative	N/A	N/A	No	N/A	N/A	Found dead	This study
266	*Zamenis longissimus*	2023-06-08	46.0	8.4	Piedmont	Subadult	No	Dry Swab (3)	Negative	N/A	N/A	No	N/A	N/A		This study
267	*Vipera berus walser*	2023-06-10	45.9	8.2	Piedmont	Adult	No	Dry Swab (3)	Negative	N/A	N/A	No	N/A	N/A		This study
268	*Vipera berus walser*	2023-06-10	45.9	8.2	Piedmont	Adult	No	Dry Swab (3)	Negative	N/A	N/A	No	N/A	N/A		This study
269	*Coronella austriaca*	2023-06-11	45.9	8.2	Piedmont	Juvenile	No	Dry Swab (3)	Positive	17	N/A	No	N/A	Oo present		This study

270	*Coronella austriaca*	2023-06-11	45.9	8.2	Piedmont	Adult	No	Dry Swab (3)	Negative	N/A	N/A	No	N/A	N/A		This study
271	*Vipera berus walser*	2023-06-11	45.9	8.2	Piedmont	Juvenile	No	Dry Swab (3)	Negative	N/A	N/A	No	N/A	N/A		This study
272	*Vipera aspis* sspp.	2023-06-11	45.9	8.2	Piedmont	Adult	No	Dry Swab (3)	Negative	N/A	N/A	No	N/A	N/A		This study
273	*Coronella austriaca*	2023-06-11	45.9	8.2	Piedmont	Adult	No	Dry Swab (3)	Negative	N/A	N/A	No	N/A	N/A		This study
274	*Hierophis viridiflavus* sspp.	2023-06-10	45.2	9.6	Lombardy	Adult	N/A	Dry Swab (3)	Negative	N/A	N/A	No	N/A	N/A	Found dead	This study
275	*Vipera aspis* sspp.	2005-07-13	44.9	7.0	Piedmont	Adult	N/A	Dry Swab (3)	Negative	N/A	N/A	No	N/A	N/A	Specimen in alcohol	This study
276	*Vipera aspis* sspp.	2023-06-13	9.2	45.9	Piedmont	Adult	N/A	Dry Swab (3)	Negative	N/A	N/A	No	N/A	N/A	Specimen in alcohol	This study
277	*Hierophis viridiflavus* sspp.	2023-06-16	46.0	8.4	Piedmont	Adult	N/A	Dry Swab (3)	Negative	N/A	N/A	No	N/A	N/A	Found dead	This study
278	*Hierophis viridiflavus* sspp.	2023-06-17	46.0	8.5	Piedmont	Adult	N/A	Dry Swab (3)	Negative	N/A	N/A	No	N/A	N/A	Found dead	This study
279	*Natrix tessellata*	2023-06-17	46.0	8.5	Piedmont	Adult	Yes	Dry Swab (3) + Tissue (in formalin)	Negative	N/A	N/A	No	N/A	N/A		This study
280	*Zamenis longissimus*	2023-06-17	46.0	8.4	Piedmont	Juvenile	No	Dry Swab (3)	Negative	N/A	N/A	No	N/A	N/A	Found dead	This study
281	*Vipera aspis* sspp.	2023-06-22	46.0	10.1	Lombardy	Adult	No	Dry Swab (3)	Negative	N/A	N/A	No	N/A	N/A		This study
282	*Vipera aspis* sspp.	2023-06-22	46.0	10.1	Lombardy	Adult	No	Dry Swab (3)	Positive	57	II	No	N/A	Oo present		This study
283	*Vipera berus marasso*	2023-06-22	46.0	10.2	Lombardy	Adult	No	Dry Swab (3) + Tissue (in formalin)	Negative	N/A	N/A	No	N/A	N/A		This study
284	*Natrix tessellata*	2023-06-25	46.0	8.4	Piedmont	Adult	No	Dry Swab (3)	Negative	N/A	N/A	No	N/A	N/A		This study
285	*Hierophis viridiflavus* sspp.	2023-06-19	45.1	9.7	Lombardy	Adult	No	Dry Swab (3)	Negative	N/A	N/A	No	N/A	N/A		This study
286	*Vipera aspis* sspp.	2023-06-27	46.3	8.3	Piedmont	Adult	No	Dry Swab (3)	Negative	N/A	N/A	No	N/A	N/A		This study
287	*Vipera aspis* sspp.	2023-06-27	46.3	8.3	Piedmont	Adult	No	Dry Swab (3)	Negative	N/A	N/A	No	N/A	N/A		This study
288	*Vipera aspis* sspp.	2023-06-27	46.3	8.3	Piedmont	Adult	No	Dry Swab (3)	Negative	N/A	N/A	No	N/A	N/A		This study
289	*Vipera aspis* sspp.	2023-06-27	45.8	10.6	Lombardy	Adult	No	Dry Swab (3)	Negative	N/A	N/A	No	N/A	N/A		This study
290	*Natrix maura*	2023-07-03	44.1	8.1	Liguria	Juvenile	No	Dry Swab (3)	Negative	N/A	N/A	No	N/A	N/A		This study

291	*Zamenis longissimus*	2023-07-04	44.0	8.1	Liguria	Adult	N/A	Dry Swab (3)	Negative	N/A	N/A	No	N/A	N/A	Found dead	This study
292	*Natrix maura*	2023-07-04	44.0	8.1	Liguria	Adult	No	Dry Swab (3)	Negative	N/A	N/A	No	N/A	N/A		This study
293	*Vipera berus walser*	2016-08-08	45.7	7.9	Aosta Valley	Adult	N/A	Dry Swab (3)	Negative	N/A	N/A	No	N/A	N/A	Specimen in alcohol	This study
294	*Coronella austriaca*	2023-07-16	46.0	8.4	Piedmont	Adult	No	Dry Swab (3)	Negative	N/A	N/A	No	N/A	N/A		This study
295	*Natrix tessellata*	2023-07-19	45.9	8.5	Piedmont	Adult	Yes	Dry Swab (3) + Tissue (in formalin)	Positive	33	II (IIDE)	Yes	Negative (inflammation)	Apparent Ophidiomycosis		This study
296	*Vipera aspis* sspp.	2023-07-22	46.0	8.4	Piedmont	Adult	No	Dry Swab (3)	Negative	N/A	N/A	No	N/A	N/A		This study
297	*Vipera aspis* sspp.	2023-07-29	46.2	9.6	Lombardy	Adult	No	Dry Swab (3)	Negative	N/A	N/A	No	N/A	N/A		This study
298	*Natrix helvetica sicula*	2023-07-29	46.2	9.6	Lombardy	Juvenile	No	Dry Swab (3)	Negative	N/A	N/A	No	N/A	N/A		This study
299	*Vipera berus marasso*	2023-07-30	46.0	9.6	Lombardy	Adult	No	Dry Swab (3)	Negative	N/A	N/A	No	N/A	N/A		This study
300	*Vipera berus marasso*	2023-07-30	46.0	9.6	Lombardy	Adult	No	Dry Swab (3)	Negative	N/A	N/A	No	N/A	N/A		This study
301	*Vipera berus marasso*	2023-07-30	46.0	9.6	Lombardy	Adult	No	Dry Swab (3)	Negative	N/A	N/A	No	N/A	N/A		This study
302	*Coronella austriaca*	2023-08-01	46.0	8.4	Piedmont	Juvenile	No	Dry Swab (3)	Negative	N/A	N/A	No	N/A	N/A		This study
303	*Natrix tessellata*	2023-08-02	45.6	8.7	Lombardy	Adult	Yes	Dry Swab (3) + Tissue (in formalin)	Positive	1744	II (IIDE)	Yes	Negative (inflammation)	Apparent Ophidiomycosis		This study
304	*Hierophis viridiflavus* sspp.	2023-08-02	45.7	8.7	Lombardy	Adult	No	Dry Swab (3)	Negative	N/A	N/A	No	N/A	N/A		This study
305	*Hierophis viridiflavus* sspp.	2023-08-02	45.7	8.7	Lombardy	Adult	No	Dry Swab (2)	Negative	N/A	N/A	No	N/A	N/A		This study
306	*Hierophis viridiflavus* sspp.	2023-08-02	45.7	8.7	Lombardy	Adult	No	Dry Swab (2)	Negative	N/A	N/A	No	N/A	N/A		This study
307	*Zamenis longissimus*	2023-08-02	45.7	8.7	Lombardy	Adult	No	Dry Swab (2)	Negative	N/A	N/A	No	N/A	N/A		This study
308	*Hierophis viridiflavus* sspp.	2023-08-04	46.0	8.4	Piedmont	Adult	Yes	Dry Swab (2) + Tissue (in formalin)	Negative	N/A	N/A	No	N/A	N/A		This study
309	*Coronella austriaca*	2023-08-07	45.9	8.2	Piedmont	Adult	No	Dry Swab (2)	Negative	N/A	N/A	No	N/A	N/A		This study
310	*Vipera aspis* sspp.	2023-08-07	45.9	8.2	Piedmont	Adult	No	Dry Swab (1)	Negative	N/A	N/A	No	N/A	N/A		This study
311	*Natrix helvetica sicula*	2023-08-08	46.0	8.3	Piedmont	Adult	No	Dry Swab (1)	Negative	N/A	N/A	No	N/A	N/A		This study

312	*Natrix helvetica sicula*	2023-08-09	46.0	8.3	Piedmont	Adult	No	Dry Swab (1)	Negative	N/A	N/A	No	N/A	N/A		This study
313	*Natrix helvetica sicula*	2023-08-09	46.0	8.3	Piedmont	Adult	No	Dry Swab (1)	Negative	N/A	N/A	No	N/A	N/A		This study
314	*Natrix helvetica sicula*	2023-08-09	46.0	8.3	Piedmont	Adult	No	Dry Swab (1)	Negative	N/A	N/A	No	N/A	N/A		This study
315	*Natrix helvetica sicula*	2023-08-09	46.0	8.3	Piedmont	Adult	No	Dry Swab (1)	Negative	N/A	N/A	No	N/A	N/A		This study
316	*Natrix helvetica sicula*	2023-08-09	46.0	8.3	Piedmont	Juvenile	No	Dry Swab (1)	Negative	N/A	N/A	No	N/A	N/A		This study
317	*Natrix helvetica sicula*	2023-08-09	46.0	8.3	Piedmont	Adult	No	Dry Swab (1)	Negative	N/A	N/A	No	N/A	N/A		This study
318	*Natrix helvetica sicula*	2023-08-09	46.0	8.3	Piedmont	Adult	No	Dry Swab (1)	Negative	N/A	N/A	No	N/A	N/A		This study
319	*Natrix helvetica sicula*	2023-08-09	46.0	8.3	Piedmont	Adult	No	Dry Swab (1)	Negative	N/A	N/A	No	N/A	N/A		This study
320	*Natrix helvetica sicula*	2023-08-09	46.0	8.3	Piedmont	Adult	No	Dry Swab (1)	Negative	N/A	N/A	No	N/A	N/A		This study
321	*Natrix helvetica sicula*	2023-08-09	46.0	8.3	Piedmont	Adult	No	Dry Swab (1)	Negative	N/A	N/A	No	N/A	N/A		This study
322	*Natrix helvetica sicula*	2023-08-09	46.0	8.3	Piedmont	Adult	No	Dry Swab (1)	Negative	N/A	N/A	No	N/A	N/A		This study
323	*Natrix helvetica sicula*	2023-08-09	46.0	8.3	Piedmont	Adult	No	Dry Swab (1)	Negative	N/A	N/A	No	N/A	N/A		This study
324	*Natrix helvetica sicula*	2023-08-21	45.7	8.7	Lombardy	Juvenile	No	Dry Swab (2)	Negative	N/A	N/A	No	N/A	N/A		This study
325	*Hierophis viridiflavus* sspp.	2023-08-21	45.7	8.7	Lombardy	Adult	No	Dry Swab (2)	Negative	N/A	N/A	No	N/A	N/A		This study
326	*Vipera ammodytes*	2023-09-09	46.3	12.4	Friuli-Venezia Giulia	Adult	Yes	Dry Swab (3) + Tissue (in formalin)	Negative	N/A	N/A	No	N/A	N/A		This study
327	*Vipera ammodytes*	2023-09-09	46.3	12.4	Friuli-Venezia Giulia	Adult	No	Dry Swab (3)	Negative	N/A	N/A	No	N/A	N/A		This study
328	*Natrix tessellata*	2023-09-12	46.0	8.5	Piedmont	Adult	Yes	Dry Swab (3)	Negative	N/A	N/A	No	N/A	N/A		This study
329	*Hierophis viridiflavus* sspp.	2023-09-13	45.2	8.2	Piedmont	Adult	No	Dry Swab (1)	Negative	N/A	N/A	No	N/A	N/A		This study
330	*Hierophis viridiflavus* sspp.	2023-09-13	45.2	8.2	Piedmont	Adult	No	Dry Swab (1)	Negative	N/A	N/A	No	N/A	N/A		This study
331	*Hierophis viridiflavus* sspp.	2023-09-13	45.2	8.2	Piedmont	Adult	No	Dry Swab (1)	Negative	N/A	N/A	No	N/A	N/A		This study
332	*Hierophis viridiflavus* sspp.	2023-09-15	46.0	8.4	Piedmont	Adult	No	Dry Swab (3)	Negative	N/A	N/A	No	N/A	N/A		This study

333	*Natrix helvetica sicula*	2023-09-19	45.7	8.6	Piedmont	Juvenile	No	Dry Swab (3)	Negative	N/A	N/A	No	N/A	N/A		This study
334	*Coronella austriaca*	2023-09-25	46.0	8.1	Piedmont	Juvenile	N/A	Dry Swab (3)	Negative	N/A	N/A	No	N/A	N/A	Found dead	This study
335	*Coronella austriaca*	2023-09-25	46.0	8.1	Piedmont	Adult	N/A	Dry Swab (3)	Negative	N/A	N/A	No	N/A	N/A	Found dead	This study
336	*Hierophis viridiflavus* sspp.	2023-09-08	46.0	8.4	Piedmont	Subadult	No	Dry Swab (3)	Negative	N/A	N/A	No	N/A	N/A		This study
337	*Hierophis viridiflavus* sspp.	2023-09-20	45.2	8.2	Piedmont	Adult	No	Dry Swab (1)	Negative	N/A	N/A	No	N/A	N/A		This study
338	*Hierophis viridiflavus* sspp.	2023-09-20	45.2	8.2	Piedmont	Adult	No	Dry Swab (1)	Negative	N/A	N/A	No	N/A	N/A		This study
339	*Hierophis viridiflavus* sspp.	2023-09-20	45.2	8.2	Piedmont	Adult	No	Dry Swab (1)	Negative	N/A	N/A	No	N/A	N/A		This study
340	*Natrix helvetica sicula*	1985-08-13	42.6	11.2	Tuscany	Adult	Yes	Museum samples (tissue in alcohol)	Positive	14	N/A	No	N/A	Apparent Ophidiomycosis	Museum sample	This study
341	*Natrix helvetica sicula*	1985 September	45.2	9.0	Lombardy	Adult	Yes	Museum samples (tissue in alcohol)	Positive	3761	I (IA)	No	N/A	Apparent Ophidiomycosis	Museum sample	This study
342	*Natrix helvetica sicula*	1985-08-14	42.6	11.2	Tuscany	Adult	Yes	Museum samples (tissue in alcohol)	Positive	18	II	No	N/A	Apparent Ophidiomycosis	Museum sample	This study
343	*Natrix helvetica sicula*	1993 June	45.7	9.3	Lombardy	Adult	Yes	Museum samples (tissue in alcohol)	Negative	N/A	N/A	No	N/A	N/A	Museum sample	This study
344	*Natrix tessellata*	1974 August	45.8	9.3	Lombardy	Adult	Yes	Museum sample (tissue in alcohol)	Positive	1325	I (IA)	No	N/A	Apparent Ophidiomycosis	Museum sample	This study
345	*Natrix tessellata*	1926 August	45.6	10.7	Veneto	Adult	Yes	Museum samples (tissue in alcohol)	Negative	N/A	N/A	No	N/A	N/A	Museum sample	This study
346	*Natrix tessellata*	1934-07-02	45.8	9.4	Lombardy	Adult	Yes	Museum samples (tissue in alcohol)	Negative	N/A	N/A	No	N/A	N/A	Museum sample	This study
347	*Natrix tessellata*	1971 N/A	45.8	9.2	Lombardy	Adult	Yes	Museum samples (tissue in alcohol)	Negative	N/A	N/A	No	N/A	N/A	Museum sample	This study
348	*Natrix tessellata*	1962-08-28	46.0	9.3	Lombardy	Adult	Yes	Museum samples (tissue in alcohol)	Negative	N/A	N/A	No	N/A	N/A	Museum sample	This study
349	*Natrix tessellata*	1963-05-09	46.0	9.3	Lombardy	Adult	Yes	Museum samples (tissue in alcohol)	Negative	N/A	N/A	No	N/A	N/A	Museum sample	This study

350	*Natrix tessellata*	1982 October	45.5	9.5	Lombardy	Adult	Yes	Museum samples (tissue in alcohol)	Negative	N/A	N/A	No	N/A	N/A	Museum sample	This study
351	*Natrix tessellata*	1985 June	45.5	9.5	Lombardy	Adult	Yes	Museum samples (tissue in alcohol)	Positive	94747	I (IB)	No	N/A	Apparent Ophidiomycosis	Museum sample	This study
352	*Natrix helvetica sicula*	1971-09-01	46.0	9.2	Lombardy	Adult	Yes	Museum samples (tissue in alcohol)	Negative	N/A	N/A	No	N/A	N/A	Museum sample	This study
353	*Natrix helvetica sicula*	1966-10-26	43.5	11.8	Tuscany	Adult	Yes	Museum samples (tissue in alcohol)	Negative	N/A	N/A	No	N/A	N/A	Museum sample	This study
354	*Natrix helvetica sicula*	1965 N/A	45.9	9.1	Lombardy	Adult	Yes	Museum samples (tissue in alcohol)	Negative	N/A	N/A	No	N/A	N/A	Museum sample	This study
355	*Natrix helvetica sicula*	1961-05-24	45.9	9.1	Lombardy	Adult	Yes	Museum samples (tissue in alcohol)	Negative	N/A	N/A	No	N/A	N/A	Museum sample	This study
356	*Natrix helvetica sicula*	1964-04-11	45.9	8.8	Lombardy	Adult	Yes	Museum samples (tissue in alcohol)	Positive	7931	I (IA)	No	N/A	Apparent Ophidiomycosis	Museum sample	This study
357	*Natrix tessellata*	2023-05-25	45.8	10.8	Veneto	Adult	No	Dry Swab (3)	Negative	N/A	N/A	No	N/A	N/A		This study
358	*Natrix tessellata*	2023-05-25	45.8	10.8	Veneto	Adult	Yes	Dry Swab (3) + Tissue (in formalin)	Negative	N/A	N/A	No	N/A	N/A		This study
359	*Natrix tessellata*	2023-05-25	45.8	10.8	Veneto	Subadult	No	Dry Swab (3)	Negative	N/A	N/A	No	N/A	N/A		This study
360	*Natrix tessellata*	2023-05-25	45.8	10.8	Veneto	Subadult	Yes	Dry Swab (3) + Tissue (in formalin)	Positive	184	II (IIDE)	Yes	Hyphae	Ophidiomycosis		This study
361	*Natrix tessellata*	2023-05-25	45.8	10.8	Veneto	Adult	No	Dry Swab (3)	Positive	31	N/A	No	N/A	Oo present		This study
362	*Natrix tessellata*	2023-05-25	45.8	10.8	Veneto	Juvenile	No	Dry Swab (3)	Negative	N/A	N/A	No	N/A	N/A		This study
363	*Natrix tessellata*	2023-06-01	46.0	11.3	Trentino-South Tyrol	Adult	No	Dry Swab (3)	Negative	N/A	N/A	No	N/A	N/A		This study
364	*Natrix tessellata*	2023-06-01	46.0	11.3	Trentino-South Tyrol	Juvenile	No	Dry Swab (3)	Negative	N/A	N/A	No	N/A	N/A		This study
365	*Natrix tessellata*	2023-06-01	46.0	11.3	Trentino-South Tyrol	Adult	No	Dry Swab (3)	Negative	N/A	N/A	No	N/A	N/A		This study
366	*Natrix tessellata*	2023-06-01	46.0	11.3	Trentino-South Tyrol	Adult	No	Dry Swab (3)	Negative	N/A	N/A	No	N/A	N/A		This study
367	*Natrix tessellata*	2023-06-01	46.0	11.3	Trentino-South Tyrol	Adult	No	Dry Swab (3)	Negative	N/A	N/A	No	N/A	N/A		This study

368	*Natrix tessellata*	2023-06-18	46.0	11.2	Trentino-South Tyrol	Adult	Yes	Dry Swab (3) + Tissue (in formalin)	Negative	N/A	N/A	No	N/A	N/A		This study
369	*Natrix tessellata*	2023-06-19	46.0	11.3	Trentino-South Tyrol	Adult	No	Dry Swab (3)	Negative	N/A	N/A	No	N/A	N/A		This study
370	*Natrix tessellata*	2023-06-19	46.0	11.3	Trentino-South Tyrol	Adult	No	Dry Swab (3)	Negative	N/A	N/A	No	N/A	N/A		This study
371	*Natrix tessellata*	2023-06-19	46.0	11.3	Trentino-South Tyrol	Adult	No	Dry Swab (3)	Negative	N/A	N/A	No	N/A	N/A		This study
372	*Natrix tessellata*	2023-06-19	46.0	11.3	Trentino-South Tyrol	Adult	No	Dry Swab (3)	Negative	N/A	N/A	No	N/A	N/A		This study
373	*Natrix tessellata*	2023-06-19	46.0	11.3	Trentino-South Tyrol	Adult	No	Dry Swab (3)	Negative	N/A	N/A	No	N/A	N/A		This study
374	*Natrix tessellata*	2023-06-19	46.0	11.3	Trentino-South Tyrol	Adult	No	Dry Swab (3)	Negative	N/A	N/A	No	N/A	N/A		This study
375	*Natrix tessellata*	2023-06-19	46.0	11.3	Trentino-South Tyrol	Adult	No	Dry Swab (3)	Negative	N/A	N/A	No	N/A	N/A		This study
376	*Natrix tessellata*	2023-06-19	46.0	11.3	Trentino-South Tyrol	Adult	No	Dry Swab (3)	Negative	N/A	N/A	No	N/A	N/A		This study
377	*Natrix tessellata*	2023-07-08	45.8	10.8	Veneto	Subadult	No	Dry Swab (3)	Positive	22	II (IIDE)	No	N/A	Oo present		This study
378	*Natrix tessellata*	2023-07-08	45.8	10.8	Veneto	Adult	No	Dry Swab (3)	Negative	N/A	N/A	No	N/A	N/A		This study
379	*Natrix tessellata*	2023-07-08	45.8	10.8	Veneto	Adult	No	Dry Swab (3)	Negative	N/A	N/A	No	N/A	N/A		This study
380	*Natrix tessellata*	2023-07-08	45.8	10.8	Veneto	Adult	No	Dry Swab (3)	Negative	N/A	N/A	No	N/A	N/A		This study
381	*Natrix tessellata*	2023-07-08	45.8	10.8	Veneto	Adult	No	Dry Swab (3)	Negative	N/A	N/A	No	N/A	N/A		This study
382	*Natrix tessellata*	2023-07-14	45.7	10.8	Veneto	Adult	Yes	Dry Swab (3) + Tissue (in formalin)	Positive	366	I & II	Yes	Hyphae	Ophidiomycosis		This study
383	*Natrix tessellata*	2023-07-14	45.7	10.8	Veneto	Adult	Yes	Dry Swab (3) + Tissue (in formalin)	Positive	28	N/A	Yes	Hyphae	Ophidiomycosis		This study
384	*Natrix helvetica cetti*	2023-08-23	39.3	9.4	Sardinia	Juvenile	No	Swab (1) in DNA stabilization solution	Negative	N/A	N/A	No	N/A	N/A		This study
385	*Natrix helvetica cetti*	2023-08-23	39.3	9.4	Sardinia	Juvenile	No	Swab (1) in DNA stabilization solution	Negative	N/A	N/A	No	N/A	N/A		This study
386	*Natrix helvetica cetti*	2023-08-23	39.3	9.4	Sardinia	Juvenile	No	Swab (1) in DNA stabilization solution	Negative	N/A	N/A	No	N/A	N/A		This study
387	*Natrix helvetica cetti*	2023-08-23	39.3	9.4	Sardinia	Juvenile	No	Swab (1) in DNA	Negative	N/A	N/A	No	N/A	N/A		This study

								stabilization solution								
388	*Natrix helvetica cetti*	2023-08-23	39.3	9.4	Sardinia	Juvenile	No	Swab (1) in DNA stabilization solution	Negative	N/A	N/A	No	N/A	N/A		This study
389	*Natrix helvetica cetti*	2023-08-23	39.3	9.4	Sardinia	Juvenile	No	Swab (1) in DNA stabilization solution	Negative	N/A	N/A	No	N/A	N/A		This study
390	*Natrix tessellata*	2023-10-23	41.6	15.9	Apulia	Adult	No	Tissue (frozen)	Negative	N/A	N/A	No	N/A	N/A	Found dead	This study
391	*Zamenis longissimus*	2023-10-23	41.6	15.9	Apulia	Adult	No	Tissue (in alcohol)	Negative	N/A	N/A	No	N/A	N/A	Found dead	This study
392	*Natrix tessellata*	2020-05-19	46.2	9.4	Lombardy	Adult	No	Dry Swab (2)	Negative	N/A	N/A	No	N/A	N/A		This study
393	*Vipera aspis* sspp.	2023-09-09	45.9	10.0	Lombardy	Adult	No	Dry Swab (2)	Negative	N/A	N/A	No	N/A	N/A		This study
394	*Hierophis viridiflavus* sspp.	2022-02-25	45.7	9.4	Lombardy	Adult	No	Dry Swab (3)	Negative	N/A	N/A	No	N/A	N/A		This study
395	*Vipera aspis* sspp.	2023-04-24	45.9	8.7	Lombardy	Adult	No	Dry Swab (2)	Negative	N/A	N/A	No	N/A	N/A		This study
396	*Hierophis viridiflavus* sspp.	2023-04-24	45.9	8.7	Lombardy	Subadult	No	Dry Swab (2)	Negative	N/A	N/A	No	N/A	N/A		This study
397	*Hierophis viridiflavus* sspp.	2023-04-24	45.9	8.7	Lombardy	Adult	N/A	Molt (frozen)	Positive	27	N/A	No	N/A	Oo present	Shed	This study
398	*Malpolon monspessulanus*	2023-05-08	44.0	8.2	Liguria	Adult	No	Dry Swab (2)	Negative	N/A	N/A	No	N/A	N/A		This study
399	*Hierophis viridiflavus* sspp.	2023-04-24	45.9	8.7	Lombardy	Juvenile	Yes	Dry Swab (3) + Tissue (in formalin)	Negative	N/A	N/A	No	N/A	N/A		This study
400	*Elaphe quatuorlineata*	2023-08-01	41.5	13.8	Lazio	Adult	No	Dry Swab (2)	Negative	N/A	N/A	No	N/A	N/A	Found dead	This study
401	*Malpolon monspessulanus*	2023-06-15	44.1	8.1	Liguria	Adult	No	Dry Swab (2)	Negative	N/A	N/A	No	N/A	N/A		This study
402	*Hierophis viridiflavus* sspp.	2023-06-24	44.9	9.3	Lombardy	Adult	N/A	Molt (frozen)	Negative	N/A	N/A	No	N/A	N/A	Shed	This study
403	*Natrix helvetica sicula*	2023-07-25	41.4	14.5	Campania	Juvenile	No	Dry Swab (2)	Negative	N/A	N/A	No	N/A	N/A		This study
404	*Vipera berus marasso*	2023-05-25	46.0	9.6	Lombardy	Juvenile	No	Dry Swab (2)	Negative	N/A	N/A	No	N/A	N/A		This study
405	*Vipera ursinii*	2023-08-06	42.4	13.7	Abruzzo	Adult	No	Dry Swab (2)	Negative	N/A	N/A	No	N/A	N/A		This study
406	*Vipera ursinii*	2023-08-06	42.4	13.7	Abruzzo	Adult	No	Dry Swab (2)	Negative	N/A	N/A	No	N/A	N/A		This study

407	*Coronella girondica*	2023-04-26	44.9	9.3	Lombardy	Adult	No	Dry Swab (2) + Tissue (in formalin)	Negative	N/A	N/A	No	N/A	N/A		This study
408	*Vipera berus marasso*	2023-05-26	46.0	9.6	Lombardy	Adult	No	Dry Swab (2)	Negative	N/A	N/A	No	N/A	N/A		This study
409	*Malpolon monspessulanus*	2023-04-08	44.1	8.2	Liguria	Juvenile	Yes	Dry Swab (2) + Tissue (in formalin)	Negative	N/A	N/A	No	N/A	N/A		This study
410	*Natrix helvetica sicula*	2023-08-10	44.4	9.3	Liguria	Juvenile	No	Dry Swab (2)	Negative	N/A	N/A	No	N/A	N/A		This study
411	*Malpolon monspessulanus*	2023-04-08	44.1	8.2	Liguria	Adult	No	Dry Swab (2)	Negative	N/A	N/A	No	N/A	N/A		This study
412	*Hierophis viridiflavus* sspp.	2023-08-10	44.5	9.3	Liguria	Adult	N/A	Dry Swab (2) + Tissue (frozen)	Negative	N/A	N/A	No	N/A	N/A	Found dead	This study
413	*Natrix helvetica sicula*	2023-10-09	45.8	9.3	Lombardy	Adult	No	Dry Swab (1)	Negative	N/A	N/A	No	N/A	N/A		This study
414	*Hierophis viridiflavus* sspp.	2022-10-09	39.2	8.6	Sardinia	Adult	No	Dry Swab (2)	Negative	N/A	N/A	No	N/A	N/A		This study
415	*Zamenis longissimus*	2020-10-22	46.1	9.6	Lombardy	Adult	No	Dry Swab (1)	Negative	N/A	N/A	No	N/A	N/A		This study
416	*Coronella austriaca*	2022-06-09	45.9	8.2	Piedmont	Adult	No	Dry Swab (1)	Negative	N/A	N/A	No	N/A	N/A		This study
417	*Coronella austriaca*	2022-06-09	45.9	8.2	Piedmont	Adult	No	Dry Swab (1)	Negative	N/A	N/A	No	N/A	N/A		This study
418	*Coronella austriaca*	2021-10-17	37.1	15.0	Sicily	Adult	No	Dry Swab (2)	Negative	N/A	N/A	No	N/A	N/A		This study
419	*Vipera aspis* sspp.	2022-06-09	45.9	8.2	Piedmont	Adult	No	Dry Swab (1)	Negative	N/A	N/A	No	N/A	N/A		This study
420	*Vipera aspis* sspp.	2022-06-09	45.9	8.2	Piedmont	Adult	No	Dry Swab (1)	Negative	N/A	N/A	No	N/A	N/A		This study
421	*Vipera aspis* sspp.	2021-10-17	37.8	15.0	Sicily	Adult	No	Dry Swab (1)	Negative	N/A	N/A	No	N/A	N/A		This study
422	*Malpolon insignitus*	2023-05-06	35.5	12.6	Sicily	Adult	No	Dry Swab (2)	Negative	N/A	N/A	No	N/A	N/A		This study
423	*Macroprotodon* cf. *cucullatus*	2023-05-05	35.5	12.6	Sicily	Adult	No	Dry Swab (3)	Negative	N/A	N/A	No	N/A	N/A		This study
424	*Malpolon insignitus*	2023-05-05	35.5	12.6	Sicily	Adult	No	Dry Swab (2)	Negative	N/A	N/A	No	N/A	N/A		This study
425	*Macroprotodon* cf. *cucullatus*	2023-05-06	35.5	12.6	Sicily	Juvenile	No	Dry Swab (2)	Negative	N/A	N/A	No	N/A	N/A		This study
426	*Macroprotodon* cf. *cucullatus*	2023-05-04	35.5	12.6	Sicily	Adult	N/A	Molt (frozen)	Negative	N/A	N/A	No	N/A	N/A	Shed	This study
427	*Malpolon insignitus*	2023-05-04	35.5	12.6	Sicily	Adult	No	Dry Swab (2)	Negative	N/A	N/A	No	N/A	N/A		This study

428	*Coronella austriaca*	2023-04-01	37.9	13.4	Sicily	Adult	N/A	Molt (frozen)	Negative	N/A	N/A	No	N/A	N/A	Shed	This study
429	*Natrix helvetica sicula*	2022-04-24	40.5	15.6	Basilicata	Adult	No	Tissue (frozen)	Negative	N/A	N/A	No	N/A	N/A	Found dead	This study
430	*Natrix helvetica sicula*	2023-07-30	40.2	16.0	Basilicata	Adult	No	Dry Swab (2)	Negative	N/A	N/A	No	N/A	N/A		This study
431	*Hierophis viridiflavus* sspp.	2022-10-12	45.0	7.3	Piedmont	Adult	N/A	Tissue (frozen)	Negative	N/A	N/A	No	N/A	N/A	Found dead	This study
432	*Hierophis viridiflavus* sspp.	2022-05-25	37.2	14.5	Sicily	Adult	N/A	Tissue (frozen)	Negative	N/A	N/A	No	N/A	N/A	Found dead	This study
433	*Hierophis viridiflavus* sspp.	2022-05-14	45.8	13.5	Friuli-Venezia Giulia	Adult	N/A	Tissue (frozen)	Negative	N/A	N/A	No	N/A	N/A	Found dead	This study
434	*Vipera aspis* sspp.	2022 May	44.9	9.5	Emilia-Romagna	Adult	N/A	Tissue (frozen)	Negative	N/A	N/A	No	N/A	N/A	Found dead	This study
435	*Coronella girondica*	2022-04-12	44.9	9.5	Emilia-Romagna	Adult	N/A	Tissue (frozen)	Negative	N/A	N/A	No	N/A	N/A	Found dead	This study
436	*Zamenis longissimus*	2022 September	45.5	10.3	Lombardy	Adult	N/A	Tissue (frozen)	Negative	N/A	N/A	No	N/A	N/A	Found dead	This study
437	*Natrix helvetica cetti*	2022 July	39.3	9.4	Sardinia	Adult	N/A	Molt (frozen)	Negative	N/A	N/A	No	N/A	N/A	Shed	This study
438	*Eryx jaculus*	2022-05-20	37.1	13.9	Sicily	Adult	N/A	Tissue (frozen)	Negative	N/A	N/A	No	N/A	N/A	Found dead	This study
439	*Vipera ursinii*	2023-08-06	42.4	13.6	Abruzzo	Adult	N/A	Molt (frozen)	Negative	N/A	N/A	No	N/A	N/A	Shed	This study
440	*Vipera ursinii*	2023-08-06	42.4	13.6	Abruzzo	Adult	N/A	Molt (frozen)	Negative	N/A	N/A	No	N/A	N/A	Shed	This study
441	*Vipera ursinii*	2023-08-06	42.4	13.6	Abruzzo	Adult	N/A	Molt (frozen)	Negative	N/A	N/A	No	N/A	N/A	Shed	This study
442	*Vipera ursinii*	2023-08-06	42.4	13.6	Abruzzo	Adult	N/A	Molt (frozen)	Negative	N/A	N/A	No	N/A	N/A	Shed	This study
443	*Vipera ursinii*	2023-08-06	42.4	13.6	Abruzzo	Adult	N/A	Molt (frozen)	Negative	N/A	N/A	No	N/A	N/A	Shed	This study
444	*Hierophis viridiflavus* sspp.	2022 September	45.5	10.3	Lombardy	Adult	N/A	Molt (frozen)	Negative	N/A	N/A	No	N/A	N/A	Shed	This study
445	*Natrix helvetica cetti*	2022 July	39.3	9.4	Sardinia	Adult	N/A	Molt (frozen)	Negative	N/A	N/A	No	N/A	N/A	Shed	This study
446	*Zamenis longissimus*	2022-06-06	45.8	13.7	Friuli-Venezia Giulia	Adult	N/A	Tissue (frozen)	Negative	N/A	N/A	No	N/A	N/A	Found dead	This study
447	*Vipera aspis* sspp.	2022-04-16	37.0	15.0	Sicily	Juvenile	N/A	Tissue (frozen)	Negative	N/A	N/A	No	N/A	N/A	Found dead	This study
448	*Hierophis viridiflavus* sspp.	2021-05-23	45.2	10.9	Veneto	Adult	N/A	Tissue (frozen)	Negative	N/A	N/A	No	N/A	N/A	Found dead	This study

449	*Natrix tessellata*	2021-07-07	45.2	10.8	Lombardy	Adult	N/A	Tissue (frozen)	Negative	N/A	N/A	No	N/A	N/A	Found dead	This study
450	*Hierophis viridiflavus* sspp.	2022-03-26	45.2	10.9	Lombardy	Adult	N/A	Tissue (frozen)	Negative	N/A	N/A	No	N/A	N/A	Found dead	This study
451	*Hierophis viridiflavus* sspp.	2021-05-29	45.2	10.8	Lombardy	Adult	N/A	Tissue (frozen)	Negative	N/A	N/A	No	N/A	N/A	Found dead	This study
452	*Vipera berus marasso*	2023-07-08	46.0	9.6	Lombardy	Adult	No	Dry Swab (2)	Negative	N/A	N/A	No	N/A	N/A		This study
453	*Elaphe quatuorlineata*	2023-06-02	40.8	14.6	Campania	Adult	No	Dry Swab (2)	Negative	N/A	N/A	No	N/A	N/A		This study
454	*Vipera ursinii*	2022-10-05	42.4	13.7	Abruzzo	Adult	No	Dry Swab (2)	Negative	N/A	N/A	No	N/A	N/A		This study
455	*Vipera berus walser*	2023-05-22	45.9	8.2	Piedmont	Adult	No	Dry Swab (2)	Negative	N/A	N/A	No	N/A	N/A		This study
456	*Vipera berus marasso*	2023-07-08	46.0	9.6	Lombardy	Adult	No	Dry Swab (2)	Negative	N/A	N/A	No	N/A	N/A		This study
457	*Elaphe quatuorlineata*	2023-05-31	40.8	14.6	Campania	Adult	No	Dry Swab (2)	Negative	N/A	N/A	No	N/A	N/A		This study
458	N/A	N/A	N/A	N/A	N/A	N/A	N/A	Dry Swab (2)	Negative	N/A	N/A	No	N/A	N/A		This study
459	*Natrix helvetica sicula*	2023-07-12	45.2	8.0	Piedmont	Adult	No	Dry Swab (2)	Negative	N/A	N/A	No	N/A	N/A		This study
460	*Vipera berus walser*	2023-05-21	45.9	8.2	Piedmont	Adult	No	Dry Swab (2)	Negative	N/A	N/A	No	N/A	N/A		This study
461	*Vipera berus marasso*	2022-09-05	46.0	9.6	Lombardy	Adult	No	Dry Swab (2)	Negative	N/A	N/A	No	N/A	N/A		This study
462	*Vipera berus marasso*	2023-07-08	46.0	9.6	Lombardy	Adult	No	Dry Swab (2)	Negative	N/A	N/A	No	N/A	N/A		This study
463	*Vipera ursinii*	2022-11-07	42.4	13.7	Abruzzo	Adult	No	Dry Swab (2)	Negative	N/A	N/A	No	N/A	N/A		This study

Table S6. List of Oo-positive snakes only, including 32 animals from the current survey and 4 from our previous studies.

ID	Taxon	Date	Lat.	Long.	Region	Age class	Gross signs	Sample type	Oo mol. detection	Copies Average of above 10	Oo Clade (subclade)	Histology	Histology report	Case classification	Notes	Refs.
14	*Natrix tessellata*	2021-03-02	45.9	10.9	Trentino-South Tyrol	Juvenile	Yes	Dry swab (3) + Tissue (in alcohol)	Positive	N/A	I	Yes	Hyphae and arthroconidia	Ophidiomycosis and Oo shedder		[12,52]
15	*Natrix tessellata*	2021-03-02	45.9	10.9	Trentino-South Tyrol	Juvenile	Yes	Dry swab (3) + Tissue (in alcohol)	Positive	N/A	N/A	Yes	Hyphae	Ophidiomycosis		[12]
16	*Natrix tessellata*	2021-03-02	45.9	10.9	Trentino-South Tyrol	Juvenile	Yes	Dry swab (3) + Tissue (in alcohol)	Positive	N/A	N/A	Yes	Hyphae and arthroconidia	Ophidiomycosis and Oo shedder		[12]
17	*Natrix tessellata*	2021-03-02	45.9	10.9	Trentino-South Tyrol	Adult	No	Dry swab (3) + Tissue (in alcohol)	Positive	N/A	N/A	No	N/A	Oo present		[12]
188	*Natrix tessellata*	2021-02-28	45.9	10.9	Trentino-South Tyrol	Adult	No	Dry Swab (1)	Positive	31	II	No	N/A	Oo present		This study
191	*Natrix tessellata*	2021-03-13	45.9	10.8	Trentino-South Tyrol	Adult	No	Dry Swab (2) + Tissue (frozen)	Positive	88	N/A	No	N/A	Oo present		This study
192	*Natrix tessellata*	2021-03-14	45.9	10.9	Trentino-South Tyrol	Juvenile	Yes	Dry Swab (2)	Positive	29	N/A	No	N/A	Apparent Ophidiomycosis		This study
193	*Natrix tessellata*	2021-03-14	45.9	10.8	Trentino-South Tyrol	Adult	No	Dry Swab (2) + Tissue (frozen)	Positive	13	II	No	N/A	Oo present		This study
194	*Natrix tessellata*	2021-03-14	45.9	10.8	Trentino-South Tyrol	Adult	Yes	Dry Swab (2) + Tissue (frozen)	Positive	72	N/A	No	N/A	Apparent Ophidiomycosis		This study
195	*Natrix tessellata*	2021-03-15	45.9	10.8	Trentino-South Tyrol	Juvenile	Yes	Dry Swab (2) + Tissue (frozen)	Positive	83	N/A	No	N/A	Apparent Ophidiomycosis		This study
196	*Natrix tessellata*	2021-03-24	45.9	10.9	Trentino-South Tyrol	Adult	No	Dry Swab (2) + Tissue (frozen)	Positive	28	II	No	N/A	Oo present		This study
199	*Natrix tessellata*	2021-03-28	45.9	10.8	Trentino-South Tyrol	Adult	Yes	Dry Swab (2)	Positive	52	II	No	N/A	Apparent Ophidiomycosis		This study
210	*Natrix tessellata*	2023-05-21	45.9	8.5	Piedmont	Subadult	Yes	Dry Swab (3) + Tissue (in formalin)	Positive	42	II (IIDE)	Yes	Negative (inflammation)	Apparent Ophidiomycosis		This study
211	*Natrix tessellata*	2023-05-21	45.9	8.5	Piedmont	Subadult	Yes	Dry Swab (3) + Tissue (in formalin)	Positive	32	II (IIDE)	Yes	Negative (inflammation)	Apparent Ophidiomycosis		This study
219	*Natrix tessellata*	2023-06-16	46.1	9.3	Lombardy	Subadult	No	Dry Swab (2)	Positive	16	N/A	No	N/A	Oo present		This study
225	*Natrix tessellata*	2023-08-02	46.1	9.3	Lombardy	Adult	No	Dry Swab (2)	Positive	31	II	No	N/A	Oo present		This study
228	*Natrix tessellata*	2023-08-30	46.1	9.3	Lombardy	Juvenile	No	Dry Swab (3)	Positive	314	II (IIDE)	No	N/A	Oo present		This study
229	*Natrix tessellata*	2023-08-30	46.1	9.3	Lombardy	Juvenile	No	Dry Swab (3)	Positive	70	II (IIDE)	No	N/A	Oo present		This study
243	*Hierophis viridiflavus* sspp.	2023-05-03	46.0	8.4	Piedmont	Adult	No	Dry Swab (3) + Tissue (in formalin)	Positive	66	II (IIDE)	Yes	Hyphae and arthroconidia	Ophidiomycosis and Oo shedder		This study

258	*Hierophis viridiflavus* sspp.	2023-05-31	46.0	8.4	Piedmont	Adult	Yes	Dry Swab (2) + Tissue (in formalin)	Positive	619	II (IIDE)	Yes	Hyphae and arthroconidia	Ophidiomycosis and Oo shedder		This study
269	*Coronella austriaca*	2023-06-11	45.9	8.2	Piedmont	Juvenile	No	Dry Swab (3)	Positive	17	N/A	No	N/A	Oo present		This study
282	*Vipera aspis* sspp.	2023-06-22	46.0	10.1	Lombardy	Adult	No	Dry Swab (3)	Positive	57	II	No	N/A	Oo present		This study
295	*Natrix tessellata*	2023-07-19	45.9	8.5	Piedmont	Adult	Yes	Dry Swab (3) + Tissue (in formalin)	Positive	33	II (IIDE)	Yes	Negative (inflammation)	Apparent Ophidiomycosis		This study
303	*Natrix tessellata*	2023-08-02	45.6	8.7	Lombardy	Adult	Yes	Dry Swab (3) + Tissue (in formalin)	Positive	1744	II (IIDE)	Yes	Negative (inflammation)	Apparent Ophidiomycosis		This study
340	*Natrix helvetica sicula*	1985-08-13	42.6	11.2	Tuscany	Adult	Yes	Museum samples (tissue in alcohol)	Positive	14	N/A	No	N/A	Apparent Ophidiomycosis	Museum sample	This study
341	*Natrix helvetica sicula*	1985 September	45.2	9.0	Lombardy	Adult	Yes	Museum samples (tissue in alcohol)	Positive	3761	I (IA)	No	N/A	Apparent Ophidiomycosis	Museum sample	This study
342	*Natrix helvetica sicula*	1985-08-14	42.6	11.2	Tuscany	Adult	Yes	Museum samples (tissue in alcohol)	Positive	18	II	No	N/A	Apparent Ophidiomycosis	Museum sample	This study
344	*Natrix tessellata*	1974 August	45.8	9.3	Lombardy	Adult	Yes	Museum sample (tissue in alcohol)	Positive	1325	I (IA)	No	N/A	Apparent Ophidiomycosis	Museum sample	This study
351	*Natrix tessellata*	1985 June	45.5	9.5	Lombardy	Adult	Yes	Museum samples (tissue in alcohol)	Positive	94747	I (IB)	No	N/A	Apparent Ophidiomycosis	Museum sample	This study
356	*Natrix helvetica sicula*	1964-04-11	45.9	8.8	Lombardy	Adult	Yes	Museum samples (tissue in alcohol)	Positive	7931	I (IA)	No	N/A	Apparent Ophidiomycosis	Museum sample	This study
360	*Natrix tessellata*	2023-05-25	45.8	10.8	Veneto	Subadult	Yes	Dry Swab (3) + Tissue (in formalin)	Positive	184	II (IIDE)	Yes	Hyphae	Ophidiomycosis		This study
361	*Natrix tessellata*	2023-05-25	45.8	10.8	Veneto	Adult	No	Dry Swab (3)	Positive	31	N/A	No	N/A	Oo present		This study
377	*Natrix tessellata*	2023-07-08	45.8	10.8	Veneto	Subadult	No	Dry Swab (3)	Positive	22	II (IIDE)	No	N/A	Oo present		This study
382	*Natrix tessellata*	2023-07-14	45.7	10.8	Veneto	Adult	Yes	Dry Swab (3) + Tissue (in formalin)	Positive	366	I & II	Yes	Hyphae	Ophidiomycosis		This study
383	*Natrix tessellata*	2023-07-14	45.7	10.8	Veneto	Adult	Yes	Dry Swab (3) + Tissue (in formalin)	Positive	28	N/A	Yes	Hyphae	Ophidiomycosis		This study
397	*Hierophis viridiflavus* sspp.	2023-04-24	45.9	8.7	Lombardy	Adult	N/A	Molt (frozen)	Positive	27	N/A	No	N/A	Oo present	Shed	This study

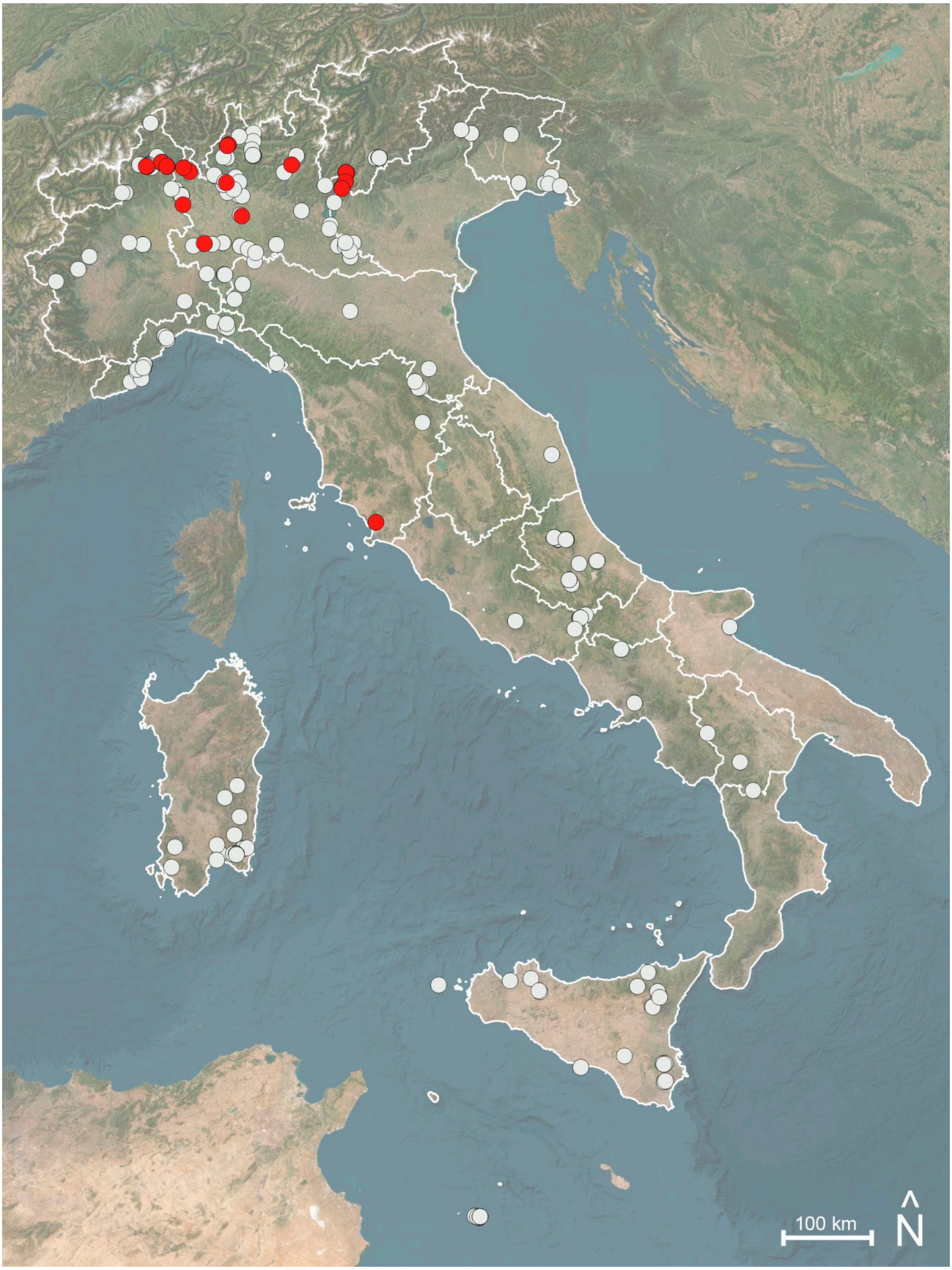

Figure S1. Map of Italy with the sampling points of the 423 snakes included in the present survey (museum samples are included). Locations with snakes negative for Oo molecular detection are marked in grey, while locations with positive snakes are marked in red. Map created using QGIS 3.28 with ESRI Satellite imagery.

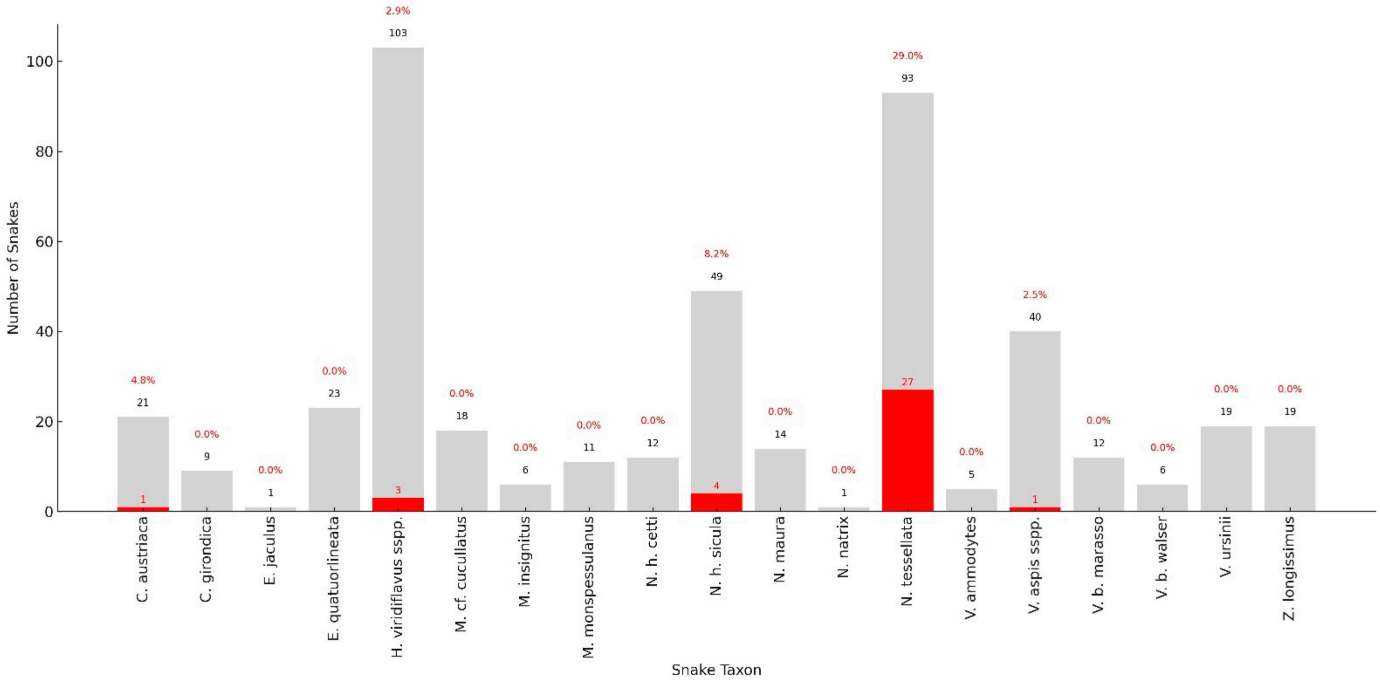

Figure S2. Number of sampled snakes (gray) and Oo-positive cases (red) by snake taxon. Museum samples and data from our previous national studies are also included.

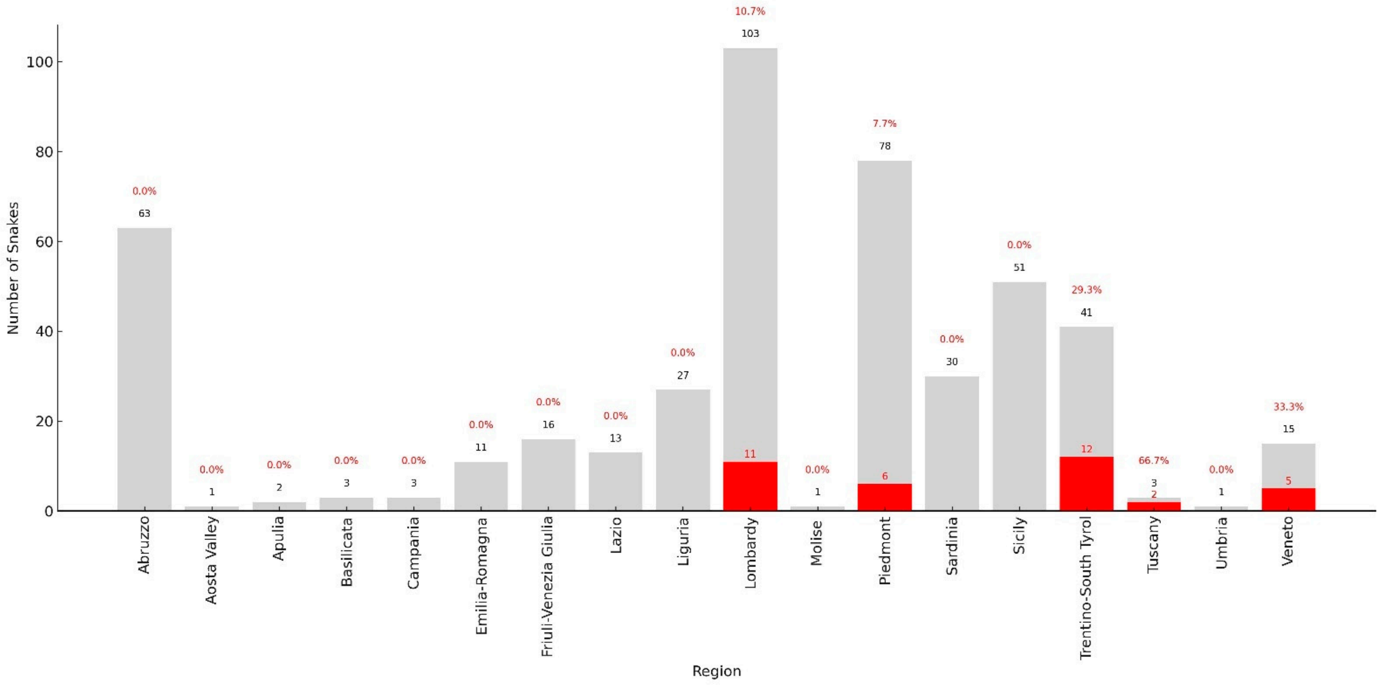

Figure S3. Number of sampled snakes (gray) and Oo-positive cases (red) by region. Museum samples and data from our previous national studies are also included.

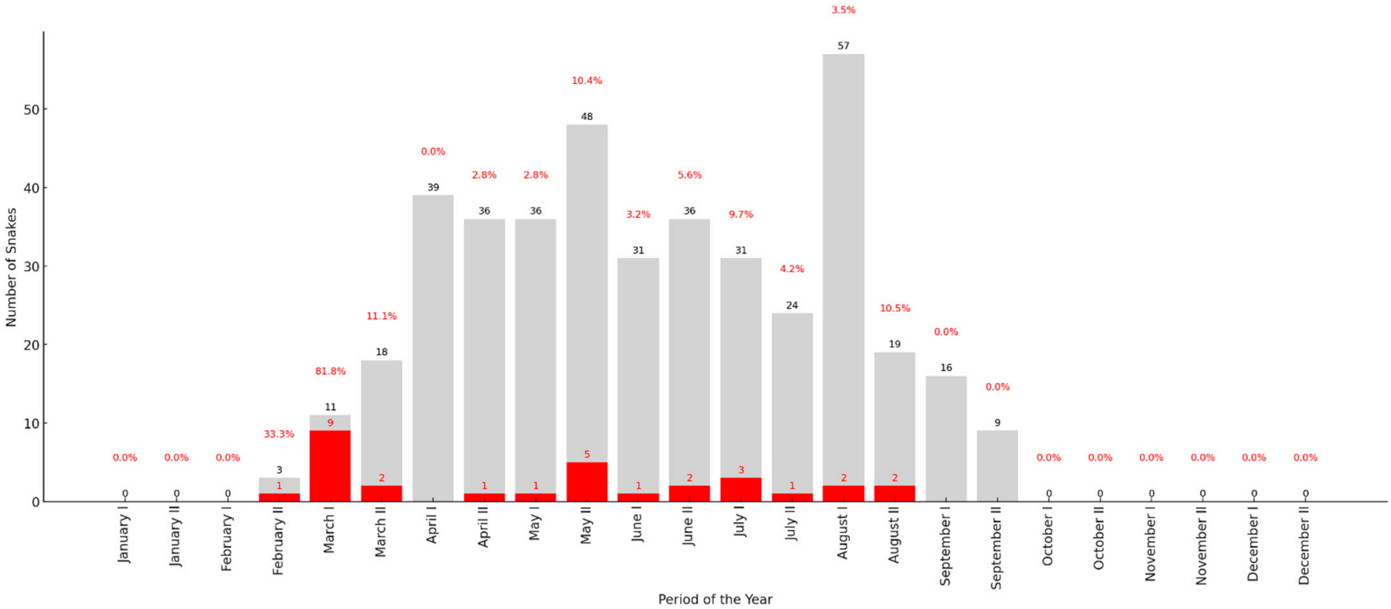

Figure S4. Number of contemporary sampled snakes (gray) and Oo-positive cases (red) by time of year. The x-axis shows the first (I) and second (II) halves of each month. Data from our previous national studies are also included.
